# Interplay Between Poly(ADP-ribosyl)ation and Specific Inner Cellular Events That Suggest Combination Strategies for Overcoming PARP Inhibitor Resistance

**DOI:** 10.3390/pharmaceutics18030355

**Published:** 2026-03-12

**Authors:** Lingwen Xu, Xiangyu Kong, Bin Zhang, Hao Ma, Xinzhi Li, Yuxiao Deng, Wentao Liu, Wenjie Ren, Xuan Tang, Daizhou Zhang

**Affiliations:** 1 Institute of Chemical Drugs, Shandong Academy of Pharmaceutical Sciences, Jinan 250101, China; kongxiangyu@sdaps.cn (X.K.); zhangbin@sdaps.cn (B.Z.); lixinzhi@sdaps.cn (X.L.); dengyuxiao@sdaps.cn (Y.D.); liuwentao@sdaps.cn (W.L.); renwenjie@sdaps.cn (W.R.); tangxuan@sdaps.cn (X.T.); 2 Shandong Engineering Research Center of Pharmaceutical Green Intelligent Technology, Shandong Academy of Pharmaceutical Sciences, Jinan 250101, China; 3 Key Laboratory of Protection, Development and Utilization of Medicinal Resources in Liupanshan Area, Ministry of Education, Ningxia Medical University, Yinchuan 750004, China; yinchuanmh@nxmu.edu.cn

**Keywords:** DNA damage response, PARP inhibitors, synthetic lethality, homologous recombination deficiency, resistance, combination therapy

## Abstract

Therapeutic resistance remains a major obstacle to durable cancer control, with functional reprogramming of the DNA damage response (DDR) network playing a central role. The poly(ADP-ribose) polymerase (PARP) family, particularly PARP1 and PARP2, is crucial for maintaining genomic integrity. By exploiting synthetic lethality, PARP inhibitors (PARPi) selectively target tumors with homologous recombination deficiency (HRD) and are integral to precision therapy in ovarian, breast, and prostate cancers. However, over 40% of patients with BRCA1/2 alterations develop resistance, and patient eligibility remains limited by the low prevalence of HRD mutations. In this review, we summarize the molecular mechanisms of PARPi action, resistance pathways, and emerging combination strategies. PARPi resistance arises through HR restoration (e.g., BRCA1/2 reversion mutations), replication fork protection, RAD51-mediated strand invasion, and metabolic reprogramming. Combination therapies, integrating PARPi with histone deacetylase inhibitors, cyclin-dependent kinase inhibitors, immune checkpoint blockade, or radiation, enhance efficacy by converging on DNA repair pathways and the tumor immune microenvironment. A deeper understanding of coordinated DDR regulation and rationally designed combination regimens will be essential for overcoming PARPi resistance and advancing adaptive, precision-based therapeutic strategies.

## 1. Introduction

**Figure 1:**
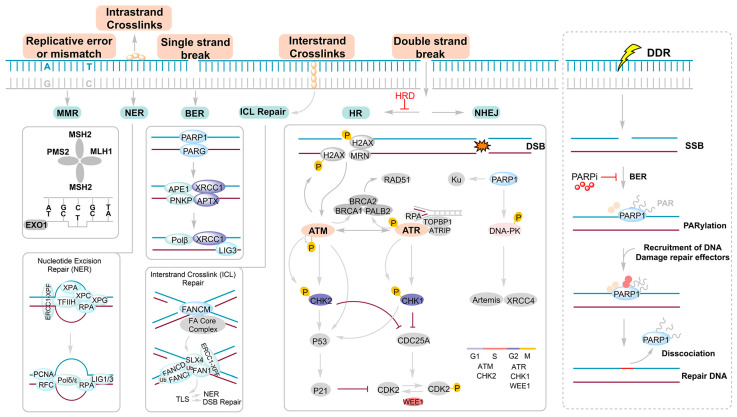
Schematic diagram of DNA-damage response (DDR) pathways and induction of synthetic lethality by PARPi. Genome integrity is continuously challenged by endogenous and exogenous genotoxic factors, including reactive oxygen species (ROS), replication stress, irradiation, and chemicals. The DDR pathways address these challenges by recognizing and repairing damage to prevent the accumulation of genome instability. These core DDR pathways—mismatch repair (MMR), nucleotide excision repair (NER), base excision repair (BER), homologous recombination repair (HRR), non-homologous end joining (NHEJ), and interstrand crosslink repair (ICLR)—collectively maintain genomic integrity. Dysregulation in any of these systems is consequently linked to genomic instability and cancer progression. When DNA SSBs occur, PARP1 is swiftly attracted to the sites of damage, where it facilitates PARylation to recruit repair proteins. In HRD cells exposed to PARPi, unrepaired lesions are converted into DSBs that are forced to undergo error-prone repair via the NHEJ pathway. Owing to the absence of template guidance, NHEJ-driven repair promotes the accumulation of genomic mismatches, leading to chromosomal instability and ultimately triggering apoptosis or mitotic catastrophe. This process underlies the therapeutic basis of PARPi-induced synthetic lethality in BRCA-mutated tumors. P, phosphorylation; gray arrows, stimulatory modifications; red flat-headed arrows, inhibitory modifications.

Resistance to anticancer therapy remains a major cause of treatment failure and poor prognosis [1,2]. Increasing evidence indicates that adaptive reprogramming of the DNA damage response (DDR) network is a central driver of this process [3,4]. The DDR safeguards genomic stability by coordinating repair of diverse lesions—including single-strand breaks (SSBs), and double-strand breaks (DSBs)—through pathways such as homologous recombination (HR), non-homologous end joining (NHEJ), and SSB repair (SSBR) (Figure 1) [5,6]. Because many chemotherapeutic agents and radiotherapy exert cytotoxicity by inducing DNA damage, tumor cells can survive by enhancing or rewiring these repair circuits [7,8]. PARP enzymes detect SSBs and initiate base excision repair (BER) via ADP-ribosylation-dependent recruitment of repair factors, whereas Ataxia Telangiectasia Mutated (ATM) and Ataxia telangiectasia and Rad3-related protein kinase (ATR) function as master DDR kinases responding to DSBs and replication stress, respectively [9]. During DSB repair, the BRCA1/2–RAD51 axis drives HR, while the p53 binding protein 1 (53BP1)–Rap1 interacting factor 1 (RIF1)–Shieldin pathway restricts end resection and favors NHEJ, thereby governing pathway choice. Dysregulation of this balance enables tumor cells to efficiently repair therapy-induced damage and develop resistance [10]. A refined understanding of DDR-mediated adaptive mechanisms is therefore essential for designing more effective therapeutic strategies (Figure 1).

The poly (ADP-ribose) polymerase (PARP) family, particularly PARP1 and PARP2, plays a central role among the core regulators of DNA repair [11,12]. These enzymes are critical for maintaining genomic stability, regulating chromatin architecture, and influencing cell fate decisions [11,13,14]. PARP1 and PARP2 primarily function as sensors of SSBs [13]. Upon detecting SSBs, these enzymes rapidly localize to the damage sites, undergo conformational activation, and catalyze poly(ADP-ribosyl)ation (PARylation) of themselves and multiple downstream substrates [15,16]. This modification establishes a specialized molecular scaffold that facilitates the recruitment of key repair factors, including X-ray repair cross-complementing group 1 (XRCC1) and DNA polymerase β, thereby orchestrating efficient SSBR [12,17,18]. The essential involvement of PARP1/2 in DNA repair, coupled with the accumulation of cytotoxic DSBs upon their inhibition, provided the mechanistic rationale for harnessing synthetic lethality as a targeted therapeutic strategy.

Synthetic lethality describes a genetic interaction in which a defect in either of two genes alone is viable, but the combination of both defects results in cell death or a severe loss of cellular fitness [19]. PARP inhibitors (PARPis) represent a landmark advance in targeted cancer therapy, constituting the first clinically approved agents to exploit synthetic lethality in tumors with DNA damage repair deficiencies [19,20]. PARPis selectively eliminate HR deficiency (HRD) tumor cells, particularly those harboring mutations in BRCA1/2, PALB2, or RAD51C, thereby reshaping the therapeutic landscape of multiple solid malignancies [21,22]. These agents have significantly improved clinical outcomes in hereditary cancers, demonstrating robust efficacy in ovarian, breast, and prostate malignancies. In addition, PARPis enhance tumor sensitivity to platinum-based chemotherapy, a synergistic interaction consistently observed in both preclinical and clinical studies, thereby supporting rational combination regimens [23]. Despite these clinical successes, the broader application of PARPi is constrained by two major challenges. First, intrinsic and acquired resistance substantially limit therapeutic efficacy. More than 40% of BRCA1/2-deficient tumors fail to respond to initial PARPi therapy, and sustained treatment frequently results in acquired resistance [21]. Second, the eligible patient population remains inherently restricted. Current regulatory approvals for PARPi are largely confined to HRD tumors, most commonly those harboring BRCA mutations. However, the prevalence of such mutations is relatively low in unselected populations (approximately 5–6%, the Mediterranean basin may be even lower), and PARPis have not yet been established as efficiency therapy for highly aggressive tumors such as glioblastoma [21].

This review examines the role of PARP in DDR and the associated drug resistance to PARPi in cancer therapy. It summarizes the molecular basis of PARPi synthetic lethality, the diverse mechanisms driving PARPi resistance, and recent advances in combination strategies designed to overcome it. Overall, this review provided a framework for optimizing precision treatment of HRD tumors, expanding the eligible patient population, and guiding the rational design of combination therapies.

## 2. Role of PARP in DDR

**Figure 2:**
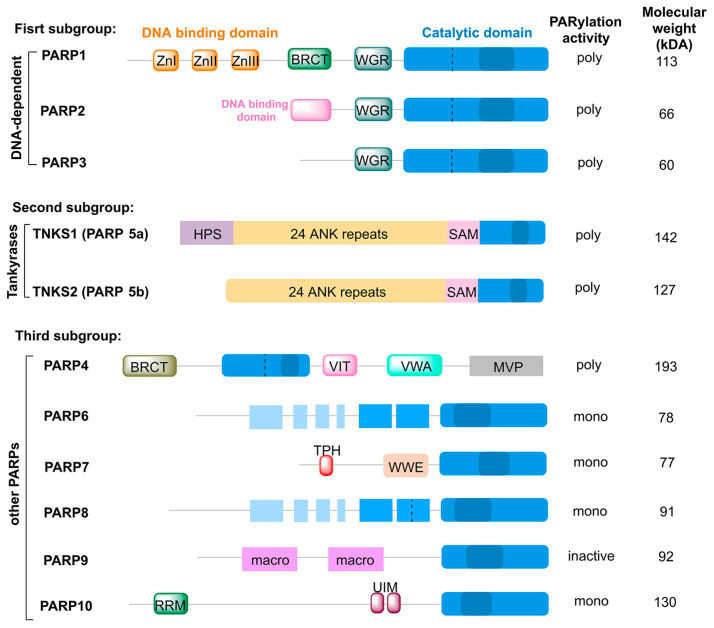
Schematic diagram of representative PARP family structures. DNA-dependent PARPs, such as PARP1, PARP2, and PARP3, feature distinct domain architectures. PARP1 contains an N-terminal DNA-binding domain, a central automodification domain, and a C-terminal catalytic domain. Its DNA-binding domain includes three zinc finger motifs, where ZnI and ZnII recognize damaged DNA and ZnIII mediates inter-domain interactions. The catalytic domain comprises a tryptophan-glycine-arginine-rich (WGR) domain, an alpha-helical domain (HD), and an ADP-ribosyltransferase (ART) domain. The tankyrase group consists of PARP5a (Tankyrase 1, TNKS1) and PARP5b (Tankyrase 2, TNKS2). Other PARPs, including PARP4, PARP6, PARP7, PARP8, PARP10, and PARP16, constitute a further category. Based on catalytic activity, PARPs are classified into two main types: mono(ADP-ribosyl)ases (MARs), which catalyze the attachment of a single ADP-ribose unit (MARylation), and poly(ADP-ribosyl)ases (PARs), which catalyze the polymerization of multiple ADP-ribose units into a chain (PARylation). Yellow region: DNA binding domain; blue region: catalytic domain; light green region: WGR domain; brown region: ankyrin domain containing 24 ankyrin repeat sequences.

The PARP superfamily encompasses a diverse group of proteins defined by a conserved catalytic domain and variable N- and C-terminal regions that confer functional specialization [24,25]. Based on biological roles, PARPs are broadly categorized into three subgroups: DNA damage repair-associated PARPs, signaling-regulatory PARPs, and functionally less characterized PARPs [26,27]. The first subgroup is exemplified by PARP1, the prototypical DDR regulator. Its N-terminal zinc finger domains recognize SSBs, initiate catalytic activation, and promote PARylation, thereby orchestrating repair factor recruitment and chromatin remodeling (Figure 2) [28,29,30]. PARP2, a structurally simpler enzyme, acts cooperatively with PARP1 in SSBR and in selected DSB repair (DSBR) contexts (Figure 2) [31,32]. PARP3, in contrast, participates more prominently in DSBR and acts as a key component of the non-homologous end joining pathway (Figure 2) [32,33].

The second subgroup, represented by tankyrases (PARP5a/5b), primarily governs cellular signaling and homeostasis rather than DNA repair (Figure 2). Through sterile alpha motif (SAM)-mediated polymerization, tankyrases regulate Wnt/β-catenin signaling, telomere maintenance, and mitotic progression [34,35]. The third subgroup consists mainly of mono-PARPs, such as PARP4, PARP6 and PARP10, which catalyze mono-ADP-ribosylation and participate in processes including cell-cycle control, stress responses, and RNA metabolism, although their precise molecular functions remain incompletely defined (Figure 2) [27]. Collectively, PARP proteins operate as an integrated signaling network rather than isolated enzymes. Elucidating both functional redundancy and context-specific specialization within this family is therefore critical for optimizing PARPi selectivity, minimizing off-target toxicity, and overcoming therapeutic resistance.

PARP-mediated cellular responses constitute a dynamic continuum that balances accurate DNA repair with controlled elimination of severely damaged cells. During the early stages of DNA damage, PARP1 is rapidly activated and promotes SSBR by catalyzing PARylation, recruiting repair factors, and facilitating chromatin remodeling to ensure repair efficiency [18,25]. In the context of DSB repair, PARP family members, including PARP1, PARP2, and PARP3, exert indirect yet pivotal control over repair outcomes by regulating repair complex assembly and pathway selection. Tumor cells can exploit this regulatory plasticity to circumvent PARPi-induced cytotoxicity through adaptive “pathway switching,” such as redirecting repair from HR to NHEJ, thereby constituting a major mechanism of therapeutic resistance [36,37]. Conversely, when DNA damage exceeds repair capacity, PARP signaling is reprogrammed from a pro-survival to a pro-death mode, prominently engaging parthanatos to eliminate cells bearing excessive genomic instability and to restrain malignant progression [19]. This context-dependent balance between DNA repair and programmed cell death is critical for preserving cellular homeostasis and ultimately dictates tumor sensitivity to DNA-damaging therapies and PARP inhibition.

The interaction between PARP and key DDR proteins, including ATM, ATR, BRCA1/2, DNA-dependent protein kinase catalytic subunit (DNA-PKcs), and 53BP1, forms a regulatory network that critically influences therapeutic response and resistance in cancer. Disruption or rewiring of this network enables tumor cells to evade the synthetic lethality induced by PARP inhibition through several interconnected mechanisms. For instance, activation of ATM or ATR signaling can stimulate CHK1/2-dependent checkpoints, prolonging the DNA repair window and facilitating DNA-PKcs-mediated NHEJ [38,39]. A major resistance mechanism involves restoration of HR in HRD tumors. BRCA1/2-mutant cancers may regain HR function through secondary mutations or epigenetic reprogramming, thereby reducing dependence on PARP activity [21,40,41]. In addition, competitive remodeling among DDR pathways contributes to resistance evolution. The chromatin regulator 53BP1 is central to this process: in BRCA1-deficient cells, 53BP1 suppresses DNA end resection and promotes NHEJ, maintaining PARPi sensitivity. However, disruption of the 53BP1 pathway—such as loss of RIF1 or REV7—relieves this restriction, restores HR, and drives resistance [42,43]. Kinase crosstalk within the DDR network also presents therapeutic opportunities. ATM-deficient tumors show increased sensitivity to PARP inhibition, supporting combined targeting of PARP and ATR [44,45]. Conversely, sustained ATR signaling has been linked to acquired PARPi resistance [46]. Collectively, these findings highlight the DDR as a dynamic and adaptive system, in which targeting key regulatory nodes, such as the 53BP1 axis and ATR checkpoint, may help overcome resistance and guide the development of rational combination therapies.

## 3. Mechanisms of Action of PARPi

### 3.1. Synthetic Lethality and Immunomodulatory Effect

The antitumor efficacy of PARPi arises from synthetic lethality, selectively targeting inherent DNA repair defects in cancer cells. This effect depends on the simultaneous impairment of two complementary repair pathways—PARP-dependent SSBR and HRR—in BRCA1/2-mutant tumors, which induces irreparable genomic instability and cell death [19,47]. Under physiological conditions, PARP family members, primarily PARP1, detect SSBs and catalyze NAD^+^-dependent PARylation to recruit BER factors like DNA polymerase β and DNA ligase III to the damage site [17,28,48]. This PARylation-driven signaling cascade coordinates the assembly of repair complexes, enabling efficient SSB resolution and genomic stability maintenance (Figure 1).

PARPis disrupt this tightly regulated balance via two principal mechanisms. By competitively binding to the enzyme’s nicotinamide adenine dinucleotide (NAD^+^) binding pocket, inhibitors such as olaparib, talazoparib, and niraparib prevent the synthesis of PAR chains [49]. This inhibition also blocks the recruitment of BER factors, which converts normally repairable SSBs into replication-associated DSBs. Second, PARPis induce “PARP trapping”, a process in which PARP1 becomes stably retained on DNA, obstructing replication fork progression and exacerbating DSB accumulation [8,50,51]. PARP trapping constitutes the dominant cytotoxic mechanism of clinically approved PARPi, with the resulting DSB burden being particularly lethal in HRD tumors. Such tumors, harboring mutations in BRCA1/2, PALB2, or RAD51C, are unable to execute efficient HR-mediated DSB repair and instead rely on error-prone NHEJ, culminating in chromosomal instability and apoptotic cell death (Figure 1) [52,53,54]. Preclinical studies have consistently demonstrated pronounced cytotoxicity of PARPi in HRD tumor cells, particularly those harboring BRCA1/2 mutations [55,56]. Clinical validation emerged from the OlympiAD trial, which showed a significant progression-free survival benefit in patients with BRCA-mutated breast cancer [57,58]. These findings confirmed the clinical applicability of synthetic lethality and supported expanding PARPi indications to other HRD malignancies, including pancreatic and prostate cancers, thereby inaugurating an era of DNA repair-guided precision oncology.

Beyond inhibiting DNA repair, PARPis also exert important immunomodulatory effects that contribute to antitumor activity. These effects arise from an integrated process involving DNA damage accumulation, immunogenic signaling, and immune regulation [21,59,60]. A central mechanism is the buildup of unresolved cytosolic DNA, which activates the cGAS–STING pathway and induces a type I interferon response independent of tumor BRCA status [59,60]. Persistent DNA lesions promote cGAS sensing of cytosolic double-stranded DNA, triggering STING activation and the expression of interferons and interferon-stimulated genes. This DNA damage-driven innate immune response enhances antigen presentation and upregulates immune checkpoint molecules such as PD-L1, thereby reshaping the tumor immune microenvironment [60,61]. Activation of the cGAS–STING axis also stimulates chemokines, including (C-C motif) ligand 5 (CCL5) and C-X-C motif chemokine ligand 10 (CXCL10), promoting CD8^+^ T-cell recruitment into the tumor microenvironment (TME) and enhancing cytotoxic T lymphocyte-mediated tumor cell killing [62,63,64]. PARPi may further amplify antitumor immunity by reducing immunosuppressive cell populations, such as regulatory T cells, thereby fostering a pro-inflammatory TME. However, these immunomodulatory effects are context-dependent. Although PARPis enhance tumor immunogenicity, they may simultaneously induce genotoxic stress in T cells, potentially impairing immune function [65,66]. Approaches such as T cell-specific PARP1 knockout have been shown to mitigate this toxicity and preserve antitumor responses [65,66]. Emerging strategies—including dual-target inhibitors (e.g., PARPi combined with histone deacetylase (HDAC) inhibitors) and rational combinations such as PARPi with cell division cycle 7 (CDC7) inhibitors—can further enhance immunomodulation by increasing cytosolic DNA accumulation, activating cGAS–STING signaling, and upregulating antigen-presentation machinery [67,68].

### 3.2. Synthetic Lethality-like Effects of PARPi in Prostate Cancer Treatment

The androgen receptor (AR) signaling pathway regulates the transcription of several DDR genes, including key HRR components such as BRCA1/2 [69]. Inhibiting this pathway with agents like enzalutamide or abiraterone downregulates these HRR genes, inducing a “BRCAness” state in tumor cells characterized by impaired HRR function similar to that caused by BRCA deficiency [70,71]. This HRR-deficient state increases tumor cell sensitivity to PARPi. PARPis exploit synthetic lethality by blocking SSBR in HRR-deficient cells, leading to the accumulation of lethal DSBs. Consequently, AR inhibition can create a therapeutic window for PARPi by inducing “BRCAness.” Recent studies demonstrate that CDK12 degradation also rapidly and significantly downregulates HRR gene expression, including BRCA1/2, thereby inducing a potent, transient “BRCAness” state. This renders even tumor cells with initially functional HRR—those lacking BRCA mutations—highly sensitive to PARPi. Acutely inducing “BRCAness” through pharmacological agents such as CDK12/7/9 degraders could therefore expand the PARPi beneficiary population beyond patients with intrinsic BRCA mutations to a broader group, including prostate cancer patients with functional HRR [72]. This strategy may also overcome acquired PARPi resistance mediated by mechanisms like BRCA reversion mutations.

### 3.3. Mechanistic Optimization of Next-Generation PARPi

PARPis, such as olaparib, niraparib, talazoparib, fluzoparib, and senaparib, have transformed the treatment landscape of HRD cancers; however, their pharmacological profiles are characterized by non-selective inhibition of both PARP1 and PARP2 [73,74,75,76,77,78,79,80]. This broad inhibitory activity, while effectively targeting PARP1, also results in unintended inhibition of PARP2. Given that PARP1 accounts for over 90% of cellular PARP catalytic activity and is essential for DDR and tumor cell survival, whereas PARP2 is required for physiological processes such as hematopoietic stem cell maintenance, its inadvertent inhibition contributes to dose-limiting toxicities, including hematological and gastrointestinal adverse effects, thereby compromising patient tolerance and clinical benefit (Table 1) [81]. Consequently, several next-generation PARPis have recently been developed to enhance target specificity, improved molecular selectivity, and minimized treatment-related toxicity (Table 1) [82,83,84,85,86,87,88,89,90,91,92,93,94,95,96,97,98].

The development of highly selective PARP1 inhibitors has become a key strategy to enhance antitumor efficacy while reducing toxicity. Structure-guided design enables preferential targeting of PARP1 over PARP2, thereby minimizing off-target inhibition. Given the critical role of PARP2 in hematopoietic stem cell maintenance, its sparing is associated with reduced myelosuppression, a broader therapeutic window, and improved feasibility of dose intensification. Molecular docking studies indicate that lead compound **33** establishes an extensive interaction network within the PARP1 catalytic site, including hydrogen bonding with Glu763 and π–π stacking interactions with Tyr889, Tyr896, and Tyr907 [81,97]. In contrast, its binding to PARP2 is weaker, with limited hydrogen bonding and restricted access to the hydrophobic pocket due to steric hindrance, explaining its marked selectivity [81,97]. Compound **34** similarly enhances PARP1 affinity through a bridging interaction involving its piperazine moiety, a coordinated water molecule, and His862 [98]. Clinically advanced agents further exemplify this strategy. Palacaparib (AZD-9574) exhibits ~500-fold selectivity, while saruparib (AZD-5305) achieves >8000-fold selectivity for PARP1, alongside favorable metabolic stability and blood–brain barrier penetration, supporting their application in BRCA-mutated breast cancer and brain metastases [81,98]. Notably, AZD5305 demonstrated superior preclinical efficacy, achieving complete responses in approximately 75% of BRCA1/2- or PALB2-mutant patient-derived xenograft models (PDX), compared with 37% for olaparib [99]. Structurally, this selectivity arises from divergence within the catalytic domains: substitution of Gly338 in PARP2 with Leu769 in PARP1 creates a unique hydrophobic pocket that confers roughly 500-fold PARP1 selectivity [99,100].

The translational advantages of these structure-optimized inhibitors have been rapidly reflected in both preclinical and clinical settings. In BRCA-mutated tumor models, saruparib induced complete responses in up to 75% of cases, markedly outperforming first-generation non-selective PARPi and extending median PFS from approximately 90 days to over 386 days [101]. These findings were corroborated in clinical studies, where saruparib demonstrated robust antitumor activity in breast cancer patients. An objective response rate (ORR) of 48.4% and a median duration of response (DOR) of 7 months were achieved at a low dose of 6 mg/day, comparable to outcomes observed at higher doses (90 mg/day; ORR 46.7%, DOR 5 months) but with a markedly improved safety profile [102,103]. Collectively, these advances signify a transition of PARPi development into an era defined by precision selectivity and therapeutic index optimization, providing a robust framework for the rational design of next-generation DNA damage-targeted anticancer agents.

## 4. Mechanisms of PARPi Resistance

Despite robust initial clinical responses, most patients ultimately develop resistance to PARPi. This resistance is mediated by four interrelated mechanisms: restoration of homologous recombination repair, most commonly via BRCA1/2 reversion mutations or functional disruption of the 53BP1–shieldin axis; re-establishment of replication fork stability through suppression of nucleolytic degradation; metabolic reprogramming that attenuates PARPi-induced cytotoxic stress; and target-level alterations, including PARP1 mutations, reactivation of PARylation capacity, or increased drug efflux mediated by transporters such as ABCB1/P-gp. These pathways frequently coexist and exhibit substantial intertumoral heterogeneity, collectively accounting for primary resistance in more than 40% of BRCA-mutant tumors and for acquired resistance during sustained treatment. In clinical practice, BRCA2 reversion mutations represent the most prevalent mechanism of PARPi resistance in ovarian, breast, and prostate cancers.

### 4.1. Reversion Mutations and HR Restoration

Restoration of HRR capacity is a central mechanism of PARPi resistance [41,104,105]. Genetically, this resistance phenotype is predominantly driven by secondary reversion mutations in HRR genes, most notably BRCA1, BRCA2, and PALB2. Clinical sequencing indicates that up to 79% of prostate cancer patients with germline BRCA or PALB2 mutations acquire such reversion mutations following PARPi treatment [41]. Approximately 60% of these frameshift reversions are flanked by microhomologous sequences, implicating DNA polymerase θ (POLQ)-mediated microhomology-mediated end joining (MMEJ) as a key mutational process. Prototypical examples include BRCA2 c.6174delT reversions, which restore the open reading frame and abrogate synthetic lethality [106,107]. In tumors with BRCA2 homozygous deletions (HomDel), resistance can alternatively emerge through the clonal selection of pre-existing BRCA2-proficient subpopulations, as single-cell transcriptomic analyses have confirmed [41,108].

Beyond genetic reversions, epigenetic reprogramming offers another mechanism for partially restoring HRR. Promoter demethylation of BRCA1 or RAD51C, for example, can reactivate gene expression and HR activity without correcting the underlying DNA sequence [109,110]. Similarly, alternative splicing variants such as BRCA1 Δ11q and Rdd-BRCA1 retain partial function and reduce PARPi (olapairb and rucaparib) sensitivity, despite not fully reconstituting canonical HR [110,111]. These adaptations allow tumors to regain sufficient HR proficiency to survive PARPi-induced DNA damage. At the pathway level, increased RAD51 focus formation acts as a functional proxy for HRR and is strongly correlated with PARPi (e.g., olaparib) resistance [41,112,113]. Disrupting HR suppressors, particularly the 53BP1-RIF1-REV7 shieldin pathway, relieves the inhibition of DNA end resection and reinstates RAD51-mediated recombination in BRCA1-deficient cells [114,115]. This restoration is further promoted by CDK-dependent phosphorylation of CtIP and the MRN complex, TOPBP1-mediated RAD51 activation, and the stabilization of RAD51 through suppression of its degradation by EMI1 or DDB2 [116,117,118]. In contrast, 53BP1 loss fails to restore HR in BRCA2-deficient tumors, highlighting the distinct mechanistic roles of BRCA1 and BRCA2 in end resection and filament stabilization [119].

**Figure 3:**
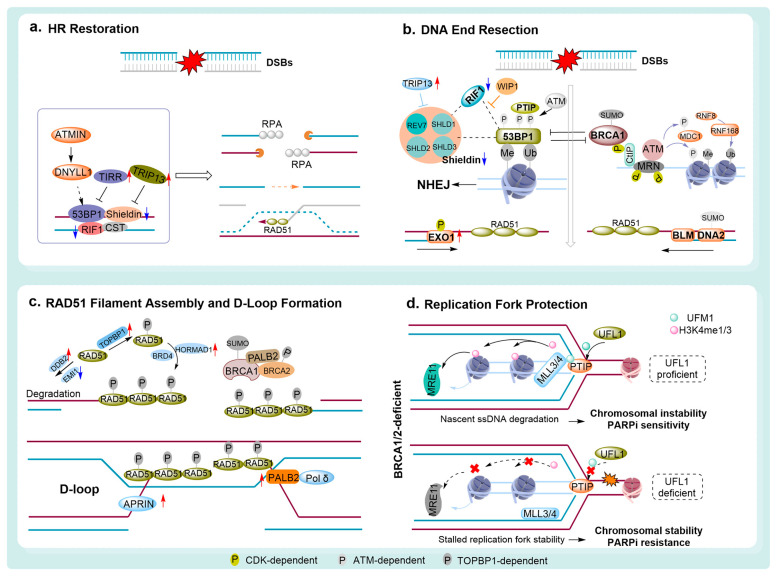
Illustration of mechanisms of PARPi resistance. (**a**) HR restoration is enabled when DYNLL1 loss, or loss of its upstream transcriptional regulator ATMIN, impairs 53BP1 oligomerization and recruitment to DSBs. Disruption of the 53BP1–RIF1–REV7–shieldin axis or the CST complex similarly compromises DNA end protection and restores end resection. In BRCA1-deficient cells, HR can be re-established by overexpressing negative regulators of NHEJ, such as TIRR, which blocks 53BP1 accumulation, or the E3 ligase TRIP13, which dismantles the REV7–shieldin complex. (**b**) DNA end resection is initiated by the MRN complex together with CtIP. MRN recruits ATM, which phosphorylates substrates including 53BP1 and MDC1. Phosphorylated MDC1 then recruits RNF8, and together with RNF168 promotes histone H2A ubiquitination. This modification, combined with H4K20 methylation, creates a binding platform for 53BP1. Activated 53BP1 engages RIF1 and PTIP, an interaction negatively regulated by WIP1, to recruit the shieldin complex, thereby suppressing resection and favoring NHEJ. BRCA1 counteracts this process to enable end resection and generate RPA-stabilized ssDNA. (**c**) RAD51 filament assembly and D-loop formation require BRCA2, which cooperates with BRCA1 via PALB2 to load RAD51 onto resected DNA. RAD51 then promotes strand invasion into a homologous template, forming a nucleoprotein filament and D-loop while preventing secondary structure formation. RAD51 stability is modulated by EMI- and DDB2-dependent degradation and TOPBP1-mediated phosphorylation, while its chromatin loading is regulated by BRD4 and HORMAD1. (**d**) Replication fork protection involves the UFM1 E3 ligase UFL1, a key regulator of fork stability and PARPi response in BRCA1/2-deficient cells. Under replication stress, UFL1 localizes to stalled forks and catalyzes PTIP UFMylation at K148, promoting assembly of the PTIP–MLL3/4 complex and deposition of H3K4me1/3 marks that recruit the MRE11 nuclease. Loss of UFL1 activity, blockade of PTIP UFMylation, or UFSP2 overexpression prevents MRE11-mediated nascent DNA degradation, thereby stabilizing replication forks and conferring PARPi resistance. P, phosphorylation; Ub, ubiquitylation; Me, methylation, SUMO, SUMOylation. red arrows, high expression; blue arrows, low expression; black arrow, stimulatory modification; black dashed arrow, translocation process.

From an evolutionary standpoint, prolonged PARPi exposure exerts a strong selective pressure that favors the expansion of clones with restored HR. The prevalence of reversion mutations differs substantially across tumor types, appearing in roughly 79% of BRCA2/PALB2-mutant prostate cancers but only 8–10% of high-grade serous ovarian cancers, with an overall frequency in ovarian cancer of about 24% [120,121,122]. This disparity likely reflects tumor-specific differences in DDR wiring and microenvironmental constraints. Importantly, HRR reactivation is not limited to BRCA reversion events; adaptive remodeling of the DDR network—including loss of 53BP1, RIF1, or shieldin components, or upregulation of HR-promoting regulators like TIRR, TRIP13, and miR-622—can independently drive resistance (Figure 3) [123,124]. The blockade of 53BP1 localization to DSBs, mediated by increased TIRR expression, also contributes to PARPi resistance in BRCA1-deficient tumors.

### 4.2. DNA End Resection

The choice of DSB repair pathway is stringently regulated by cell cycle-dependent control of DNA end resection [125,126]. During G1 phase, the recruitment of 53BP1 and RIF1 to DSBs suppresses BRCA1 loading and end resection, thereby favoring NHEJ [114,125,127]. Upon S/G2 entry, cyclin-dependent kinase (CDK)-dependent phosphorylation of the MRN complex and CtIP activates resection, shifting repair toward HR (Figure 3) [128]. Disruption of this regulatory switch drives PARPi resistance. Altered CDK signaling profoundly modulates the PARPi response, as CDK5 depletion sensitizes tumor cells to veliparib in breast cancer patients [129,130], while loss of CDK12 impairs HR and enhances olaparib sensitivity in high-grade serous ovarian cancer and triple-negative breast cancer (TNBC), irrespective of BRCA status [131,132,133]. Conversely, CDK18 promotes HR proficiency and olaparib resistance by facilitating ATR activation in glioblastoma stem-like cells [134]. Clinically, combined inhibition of PARP and CDK4/6 (e.g., dual inhibitor ZC-22, or combination therapy (olaparib + palbociclib)) has demonstrated superior efficacy to olaparib monotherapy in BRCA-mutant, estrogen receptor-positive breast cancer [135,136], underscoring the clinical relevance of CDK-mediated resection control.

**Figure 4:**
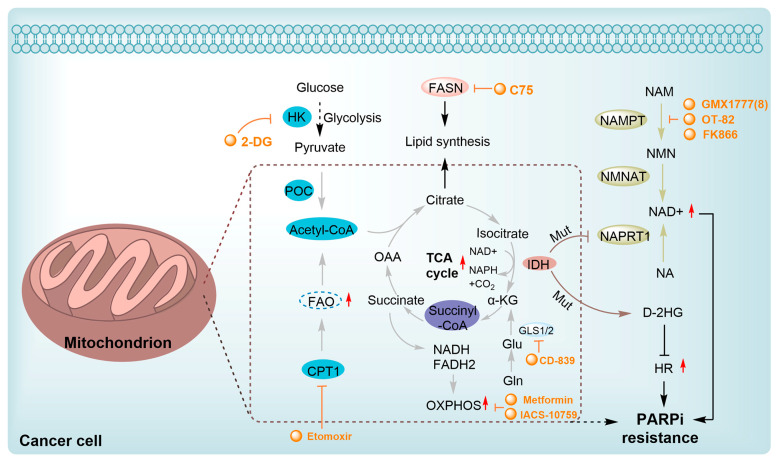
Metabolic reprogramming drives resistance to PARPi and informs corresponding metabolic intervention strategies. Tumor cells develop PARPi resistance by reprogramming their metabolism, typically through upregulating glycolysis, enhancing oxidative phosphorylation (OXPHOS), and dysregulating lipid metabolism. These adaptations substantially decrease tumor sensitivity to PARPi. Metabolic interventions to counteract this resistance include inhibiting glycolysis with 2-Deoxy-D-glucose (2-DG) and suppressing OXPHOS with agents like metformin or IACS-10759. Lipid metabolism can be targeted using C75 or etomoxir to modulate fatty acid oxidation, while NAD^+^ biosynthesis is blocked by FK866, OT-82, or GMX1777. Tumors with isocitrate dehydrogenase (IDH) mutations present additional metabolic vulnerabilities, including NAPRT1 downregulation that impairs the NAD^+^ salvage pathway. These cells also accumulate the oncometabolite D-2-hydroxyglutarate (D-2HG), which inhibits histone demethylase activity and thereby compromises homologous recombination repair. The described metabolic interventions can sensitize tumors to PARPi combination therapy. Dashed arrow, glycolysis; Red arrow, high expression; Brown arrow, mutation; black arrow, stimulatory modification; flat arrow, inhibitory modificaition.At the chromatin level, the 53BP1–RIF1–REV7 axis constitutes a dominant barrier to DNA end resection. By recognizing H4K20me2 and H2AK15ub marks, 53BP1 restricts CtIP access at DSBs, thereby limiting nuclease engagement [137,138]. Loss of 53BP1 restores end resection and HR, conferring PARPi (e.g., olaparib, talazoparib, and simmiparib) resistance across breast cancer, ovarian cancer, and glioblastoma models [139,140,141]. Mechanistically, 53BP1 and RIF1 recruit the shieldin complex (SHLD1–3 and REV7), which enforces NHEJ and maintains PARPi sensitivity [142,143,144]. Disruption of shieldin integrity, including TRIP13-mediated REV7 inactivation, promotes HR reactivation and resistance [124,145]. This pathway is further modulated by ATM-dependent phosphorylation of 53BP1, which is required for effective RIF1 and PTIP recruitment [146,147]. Accordingly, ATM deficiency enhances olaparib or niraparib sensitivity, and dual ATM–PARP inhibition yields synergistic antitumor effects in BC and Lung Adenocarcinoma [148,149]. Conversely, WIP1-mediated dephosphorylation of 53BP1 weakens end protection and promotes resistance [150].

End resection is also regulated at the enzymatic level by nucleases such as MRE11, DNA2, and EXO1 [151,152,153,154,155]. While MRE11 initiates resection, DNA2 and EXO1 mediate long- and short-range processing, collectively determining HR efficiency and therapeutic response (Figure 3) [153,154]. Pharmacological inhibition of MRE11 markedly enhances PARPi efficacy, particularly in BRCA-deficient settings [156], whereas EXO1 loss compromises HR and increases PARPi sensitivity [155]. These findings establish nuclease-mediated end resection as a central and therapeutically actionable determinant of PARPi resistance. In summary, PARPi resistance arises from convergent genetic, epigenetic, and pathway-level mechanisms that restore DNA end resection and RAD51-dependent HRR, highlighting multiple actionable vulnerabilities for therapeutic intervention.

### 4.3. RAD51 Filament Assembly and D-Loop Formation

RAD51–ssDNA nucleoprotein filaments are indispensable for HR, mediating homology search, strand invasion, and D-loop formation to preserve genomic integrity [157,158]. Accordingly, RAD51 nuclear foci have emerged as a robust functional readout of HR competence, often outperforming static BRCA mutation status in predicting response and resistance to PARPi [41,159,160,161]. The stability and dynamics of RAD51 filaments are tightly regulated. EMI1 has been identified as a key negative regulator of RAD51 through proteasomal degradation; its downregulation promotes RAD51 accumulation, restores HR in BRCA1-deficient TNBC, and confers olaparib resistance [162,163]. Similarly, the DNA damage sensor DDB2 modulates RAD51 stability via direct interaction, and DDB2 suppression induces RAD51 ubiquitination, HR deficiency, and enhanced PARPi sensitivity (Figure 3) [164]. At the post-translational level, TOPBP1-dependent phosphorylation of RAD51 at Ser14 is required for chromatin loading and focus formation; TOPBP1 loss impairs HR and sensitizes ovarian cancer cells to olaparib (Figure 3) [165,166].

Epigenetic regulation further contributes to RAD51-mediated resistance. BRD4 inhibition promotes RAD51 accumulation independently of ATM/ATR signaling, suggesting a noncanonical route to HR restoration (Figure 3) [167]. Given frequent BRD4 amplification in ovarian cancer and colorectal cancer, BRD4 inhibitors **AZD5153** represent a rational strategy to enhance olaparib efficacy [168,169,170]. In lung adenocarcinoma, aberrant HORMAD1 expression correlates with poor prognosis and drives PARPi resistance by promoting HR gene enrichment and RAD51 filament formation [171,172]. PDS5 cohesin-associated factor B (APRIN) interacts with the cohesin complex and the tumor suppressor BRCA2. It functions as a novel mediator of HR by antagonizing the inhibitory effect of RPA on RAD51 loading onto ssDNA. In ovarian cancer patients, lower APRIN expression correlates with improved survival. Furthermore, knock-down of APRIN in a zebrafish xenograft model increases cellular sensitivity to olaparib [173]. Moreover, the requirement of DNA polymerase δ (POLQ) for D-loop extension identifies an additional vulnerability that may be exploited to counteract PARPi resistance [174].

### 4.4. Replication Fork Protection

Accumulating evidence indicates that, in BRCA-mutated tumors, resistance to PARPi is frequently driven by restoration of DNA replication fork protection rather than reactivation of HR, thereby challenging the classical HR-centric model of synthetic lethality [175,176]. Reduced EZH2 expression lowers H3K27 methylation, compromises MUS81 recruitment to stalled replication forks, and thereby stabilizes forks, a mechanism sufficient to confer rucaparib resistance in BRCA2-deficient but not BRCA1-null cells [177]. Concordantly, in ovarian cancer, loss of PTIP, MLL3/4, or CHD4 prevents the loading of MRE11 at stalled replication forks, suppresses nascent strand degradation, and promotes tolerance to the PARPi **AZD2461** without restoring HR (Figure 3) [178].

FANCD2 has emerged as a central fork-protective factor that restrains RAD51-dependent, MRE11-mediated fork degradation [179]. Clinically, FANCD2 is frequently overexpressed in BRCA1/2-mutated breast, ovarian, and endometrial cancers, where its upregulation correlates with PARPi resistance [180,181,182]. Collectively, these findings establish replication fork protection as a dominant, HR-independent determinant of PARPi resistance. Fork stability is coordinately regulated by PARP1 and BRCA1/2 under replication stress. PARP1 promotes the accumulation of reversed forks to prevent aberrant restart and replication-associated DSBs [183,184], whereas BRCA1/2 shield nascent DNA from MRE11/DNA2-mediated degradation [99]. Upon PARPi treatment, PARP1 trapping exacerbates fork stalling, rendering tumor cells highly dependent on intact BRCA1/2-mediated fork protection; disruption of this axis precipitates fork collapse and cell death [177].

Additional modulators of fork dynamics further shape PARPi responses. SNF2-family chromatin remodelers (SMARCAL1, ZRANB3, HLTF) remodel stalled forks and influence therapeutic sensitivity [185,186]. Although SMARCAL1-mediated fork reversal limits ssDNA gap accumulation, it generates MRE11-sensitive intermediates; accordingly, SMARCAL1 depletion enhances resistance to PARPi and cisplatin [187,188]. Loss of RADX restores fork protection by modulating RAD51 dynamics without reactivating HR, thereby conferring PARPi resistance in BRCA2-mutant cells [188]. Moreover, UFL1-mediated UFMylation of PTIP accelerates nascent strand degradation under replication stress, whereas suppression of this pathway stabilizes forks and promotes resistance (Figure 3) [189,190]. Notably, PARPi treatment induces histone eviction, revealing a selective vulnerability in resistant cells that depend on histone homeostasis to sustain elevated replication stress [191]. Genome-wide screening identified NASP as a critical histone chaperone required for fork progression; NASP depletion markedly sensitizes tumors to PARPi by inducing replication-associated DNA damage in vitro and in vivo [191]. Mechanistically, NASP cooperates with the INO80 complex and PARP1 to maintain histone turnover and prevent catastrophic genome instability [191].

### 4.5. Metabolic Pathways

Tumor cells develop resistance to PARPi upregulating glycolysis, a phenomenon known as the Warburg effect [192,193]. The resulting lactate accumulation induces intracellular acidosis, which promotes DNA damage and exacerbates replication stress [194,195]. Enhanced glycolytic flux also reduces the cancer cell’s reliance on oxidative phosphorylation and associated DNA repair mechanisms, such as NAD^+^ regeneration, a process crucial for PARP activity [195]. Glycolytic inhibitors like 2-deoxy-D-glucose (2-DG) can therefore directly interfere with PARP activity by blocking NAD^+^ regeneration, thereby sensitizing cells to PARPi (Figure 4) [196]. Key glycolytic enzymes, including hexokinase 2, represent additional therapeutic targets [196]. Other inhibitors, such as 3-bromopyruvate (3-BrPA), have demonstrated significant efficacy in models of hepatocellular and pancreatic carcinoma (Figure 4) [195,197,198].

Nicotinamide phosphoribosyltransferase (NAMPT) is the rate-limiting enzyme in the NAD^+^ salvage pathway [194,199]. Its inhibition enhances the cytotoxicity of PARPi in tumors like TNBC [200]. Combining NAMPT inhibitors such as FK866, OT-82, and GMX1777(8) with PARPi has improved therapeutic outcomes in various cancer models (Figure 4) [192,201,202,203,204]. Among these, OT-82 exhibits potent antitumor activity in hematologic malignancies, while GMX1778 shows synergistic effects with PARPi in solid tumors (Figure 4). Mitochondrial metabolism exerts a dual influence on PARPi resistance. Agents like metformin or IACS-10759, a mitochondrial complex I inhibitor, can deplete NAD^+^ and impair DNA repair by inhibiting oxidative phosphorylation (OXPHOS) [204,205]. This inhibition renders cells more dependent on PARP-mediated repair, thereby increasing their sensitivity to PARPi. Furthermore, OXPHOS inhibition can exacerbate oxidative stress, further compromising cancer cell viability. For tumors with high oxidative capacity that develop resistance, combining mitochondrial-targeted therapies with PARPi may yield synergistic effects.

Fatty acid oxidation (FAO) and lipogenesis are crucial for maintaining membrane integrity and mitochondrial function. Enhanced FAO supplies tumor cells with additional acetyl-CoA and NADPH, which facilitate DNA repair and counteract PARPi toxicity [206]. Inhibitors of lipid metabolism, such as the fatty acid synthase inhibitor C75 or the carnitine palmitoyltransferase I inhibitor etomoxir, can impair lipid biosynthesis and mitochondrial function, leading to increased DNA damage and enhanced PARPi sensitivity (Figure 4) [206,207]. Neomorphic isocitrate dehydrogenase (IDH)-mutant tumors exhibit reduced NAD^+^ levels due to NAPRT1 downregulation and demonstrate heightened sensitivity to NAD^+^ depletion [208]. The accumulation of the oncometabolite D-2-hydroxyglutarate (D-2HG) in these tumors can also inhibit homologous recombination repair, creating a dual metabolic vulnerability [209,210]. Preclinical studies are exploring combination strategies involving PARPi like olaparib or niraparib with metformin and 2-DG to achieve synthetic lethality in HRD tumors (Figure 4).

Glycolytic reprogramming, OXPHOS dependency, adaptive lipid metabolism, and oncometabolite-driven vulnerabilities collectively provide a rationale for combination targeting strategies [192]. Future research should focus on using metabolic profiling of tumor-specific glycolytic flux, OXPHOS activity, or lipid metabolic signatures to guide the precise selection of PARPi combination regimens. The TME also fosters a protective niche for drug resistance through metabolites released by stromal cells. Targeting lactate transport or pH regulatory mechanisms may disrupt this environment and restore drug sensitivity. Advances in non-invasive metabolic biomarkers will facilitate real-time monitoring of treatment response, while emerging immunometabolic approaches, which simultaneously target resistance mechanisms and immunosuppression, offer novel strategies for overcoming PARPi resistance.

### 4.6. Increased Drug Efflux and Decreased PARP Trapping

Enhanced drug efflux mediated by ATP-binding cassette (ABC) transporters is a well-established mechanism of resistance to PARPi [211]. Key transporters—including ABCB1 (P-glycoprotein), ABCC1 (MRP1), and ABCG2 (BCRP)—actively export PARPi from tumor cells, reducing intracellular drug accumulation and limiting therapeutic efficacy. Preclinical studies in BRCA1/2-deficient mouse models have shown that acquired resistance to olaparib frequently involves strong upregulation of ABCB1, a phenotype that can be reversed by the ABCB1 inhibitor tariquidar, providing direct evidence for efflux-driven resistance [212,213]. Clinical data further support this mechanism. Recurrent genomic rearrangements involving SLC25A40–ABCB1 fusions have been identified in subsets of high-grade serous ovarian and metastatic breast cancers, linking ABCB1 activation to human disease [214,215]. Epigenetic regulation also contributes to this process: the PARP1–DOT1L–PLCG2/ABCB1 axis promotes ABCB1 transcription via DOT1L-mediated H3K79 methylation at its promoter [216]. Consistently, PARPi-resistant tumors exhibit markedly elevated *ABCB1* mRNA levels compared with sensitive counterparts. In addition to *ABCB1*, overexpression of *ABCC1* and *ABCG2* can reduce the cytotoxic activity of talazoparib in ovarian cancer models [217]. ABCB1-mediated resistance is frequently associated with cross-resistance to taxanes and anthracyclines. However, certain PARPi, such as veliparib and niraparib, are relatively poor ABCB1 substrates and may partially circumvent this mechanism [218]. Collectively, these findings highlight drug efflux as a clinically relevant yet heterogeneous resistance pathway and underscore the importance of transporter selectivity when optimizing PARPi-based therapies, particularly in heavily pretreated tumors.

Reduced PARP trapping constitutes a distinct and clinically relevant mechanism of resistance to PARPi [219]. This attenuation of trapping can arise through multiple, non-mutually exclusive mechanisms. Downregulation of PARP1 expression limits the pool of enzyme available for DNA binding, while mutations within PARP1, particularly those affecting its DNA-binding domains, impair damage recognition and stable chromatin association. PARP1 mutations, such as p.R591C, described in PARPi-resistant tumor samples, are associated with reduced PARP1 trapping activity on DNA [220]. Furthermore, the poly(ADP-ribose) glycohydrolase (PARG) enzyme is an integral component of PARP1 trapping, as it counteracts PARP1 activity by degrading PAR chains, the product of PARP activity. Since PARPis do not completely block PARP activity, loss of PARG function is sufficient to restore PAR formation and rescue downstream PARP1 signaling [221]. In BRCA2-mutant mouse mammary tumors, it has been demonstrated that loss of PARG expression allows PARylation to persist under PARP inhibition, which may contribute to drug resistance [221]. Collectively, these findings highlight reduced PARP trapping as a multifactorial resistance mechanism that directly compromises the cytotoxic foundation of PARP inhibition.

## 5. Combination Therapy Strategies for Overcoming PARPi Resistance

**Figure 5:**
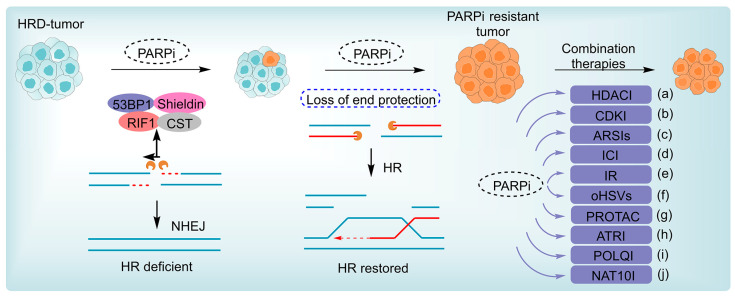
Exploiting acquired vulnerabilities in PARPi-resistant tumors: an emerging therapeutic strategy. Resistance to PARPi often develops through the loss of key DNA damage response proteins, leading to the reprogramming of repair pathways and the emergence of new, targetable vulnerabilities. Combination therapies, particularly those involving PARPi in conjunction with histone deacetylase inhibitors (HDACI), cyclin-dependent kinase inhibitors (CDKI), androgen receptor signaling inhibitors (ARSIs), immune checkpoint inhibitors (ICI), ionizing radiation (IR), oncolytic herpes simplex viruses (oHSVs), proteolysis-targeting chimeras (PROTAC), Ataxia telangiectasia and Rad3-related protein kinase inhibitors (ATRI), DNA polymerase theta inhibitors (POLQI), and N-acetyltransferase 10 inhibitors (NAT10I), have demonstrated substantial synergistic effects, offering promising avenues for overcoming resistance and enhancing therapeutic efficacy. Black arrow, stimulatory modification; Purple arrow, combined therapy.

Combination strategies are guided by the principle of pathway synergy to maximize therapeutic efficacy while maintaining acceptable toxicity, leveraging complementary mechanisms to overcome PARPi resistance and improve clinical outcomes. In terms of pathway synergy, key mechanisms include the cross-regulation of critical signaling pathways and cooperative interference with DNA damage repair networks. The additive effects of efficacy exhibit differential characteristics across various molecular backgrounds, necessitating the precise selection of treatment regimens based on the tumor’s HRR status. In overcoming PARPi resistance, novel drug design mainly focuses on the optimization of multi-target drugs and combination therapies, as well as the development of highly selective inhibitors (Figure 5) (Table 2). Compared to the complexities of drug–drug interactions, toxicity accumulation, and pharmacokinetic mismatches that may arise with combination therapies, multitarget inhibitors offer significant advantages by integrating the inhibition of two targets into a single molecule, thus reducing systemic exposure differences and minimizing toxicity accumulation.

### 5.1. Multi-Target Combination Overcoming PARPi Reisistance

#### 5.1.1. PARPi and Histone Deacetylase Inhibitors (HDACI) Combination

**Figure 6:**
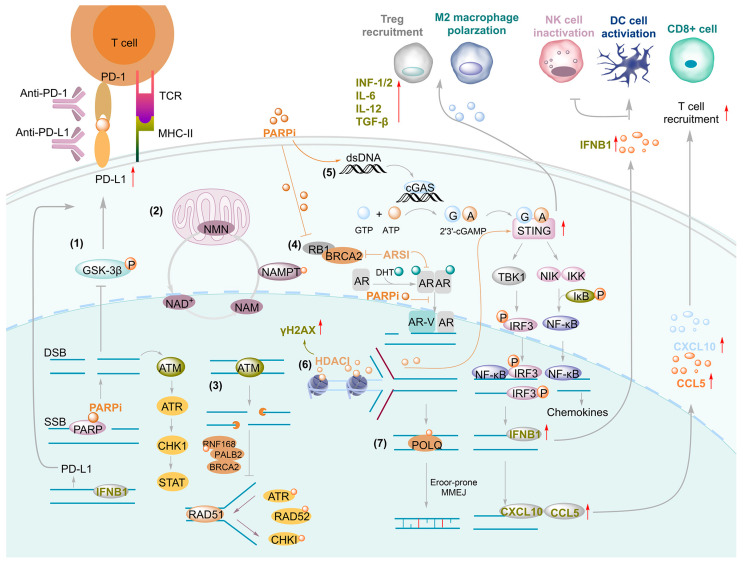
Synergistic mechanisms of PARPi combination therapies. (1) Immune checkpoint modulation: PARPi-induced DNA damage signaling suppresses GSK-3β activity, thereby elevating PD-L1 expression on tumor cells and creating a permissive context for PD-1/PD-L1 blockade to reactivate immunity within the tumor microenvironment. (2) NAD^+^ depletion-mediated synthetic lethality: Inhibiting NAMPT, the rate-limiting enzyme in the NAD^+^ salvage pathway, depletes cellular NAD^+^ pools and impairs PARP-dependent DNA repair; this metabolic vulnerability amplifies PARPi-induced genomic instability and selective tumor cell death. (3) Reversal of HRR in resistant tumors: In BRCA1-deficient contexts, HR reactivation is driven by ATM and RNF168; pharmacologic disruption of these effectors or their epigenetic regulators restores synthetic lethality and overcomes acquired PARPi resistance. (4) AR suppression-induced BRCAness: ARSI suppress HRR gene transcription while enhancing PARP activity, phenocopying BRCA loss in prostate cancer; concurrently, PARPi-induced DSB inhibit AR transcriptional function, counteracting resistance mediated by RB1/BRCA2 co-deletion or AR-V7 splice variants. (5) cGAS-STING activation and innate immune priming: Cytosolic dsDNA accumulation resulting from PARP inhibition activates cGAS, initiating STING-dependent IFN-I secretion; this cascade promotes dendritic cell maturation, cytotoxic T-cell priming, and chemokine-driven T-cell infiltration, thereby converting localized DNA damage into systemic antitumor immunity. (6) Epigenetic amplification of immune sensing: HDACi de-represses the STING promoter, restoring its expression in epigenetically silenced tumor cells and synergizing with PARPi-induced γH2AX foci to potentiate cGAS-STING signaling, which enhances immunogenic cell death and immune recognition. (7) Targeting compensatory repair and replication fork protection: Inhibition of microhomology-mediated end joining via POLQ blockade, combined with suppression of ATR or RAD52, ablates backup DNA repair and destabilizes stalled replication forks; this dual strategy prevents the survival of PARPi-resistant clones by eliminating key escape pathways. P, phosphorylation; G, GTP; A, ATP.

HDACi remodel chromatin architecture by suppressing histone deacetylase activity, thereby inducing broad transcriptional reprogramming. A pivotal consequence of this epigenetic modulation is the downregulation of key HRR genes, including BRCA1 and RAD51, resulting in attenuation of HRR capacity. Consequently, tumor cells that are initially HRR-proficient (i.e., BRCA wild-type) may acquire a functional phenotype resembling HRR deficiency, commonly referred to as “BRCAness”. The central mechanism by which HDACi overcome resistance to PARPi lies in their capacity to induce pharmacologic BRCAness and to suppress key components of the DDR, thereby synergizing with or sensitizing tumors to PARPi [222,223,224,225,226,227,228,229,230]. Specifically, HDACi such as vorinostat, quisinostat, and valproic acid (VPA) inhibit histone deacetylases, leading to chromatin relaxation and functional alterations of non-histone proteins. This epigenetic reprogramming results in the downregulation of critical HR factors, including RAD51, BRCA1/2, and FANCD2, and may also impair NHEJ regulators such as Ku80 and 53BP1. The broad attenuation of DNA repair capacity effectively creates a BRCA-like state in HR-proficient cancer cells, shifting their reliance toward error-prone alternative repair pathways, such as alt-NHEJ. Consequently, tumor cells that are intrinsically insensitive or have acquired resistance to PARPi become vulnerable to PARP inhibition through restored synthetic lethality or strong pharmacologic synergy. Notably, this sensitization is not limited to PARPi but extends to other DNA-damaging agents, including platinum compounds (e.g., cisplatin) and alkylating agents (e.g., dacarbazine, DTIC), as HDACi concurrently compromise the repair of replication-associated DNA double-strand breaks and fork collapse induced by these therapies. In addition, dual-target inhibitors of PARP and HDAC induce a BRCAness phenotype (mimicking BRCA-deficient states) and activate the cGAS-STING immune pathway, demonstrating synergistic potential in reversing resistance in both in vitro and in vivo models (Figure 6) [222,223].

Robust synergistic antitumor activity has been consistently demonstrated in vitro [224,225,226,227,228]. In Ewing sarcoma models, the bifunctional single-molecule PARP–HDAC inhibitor kt-3283 suppressed cell viability at concentrations 30–80 fold lower than olaparib or vorinostat alone, achieving EC_50_ values as low as 16.3–53 nM [224]. This enhanced potency was accompanied by pronounced S/G2–M cell-cycle arrest, increased DNA damage signaling (γH_2_AX foci formation and comet tail moments), and augmented apoptosis (Figure 6) [224,225]. Superior antitumor effects were also observed in 3D spheroid cultures and ex vivo lung metastasis models. Similarly, in small cell lung cancer (SCLC) and urothelial carcinoma cells, HDACi such as CUDC-907 or quisinostat synergized with olaparib or talazoparib (CI < 1), resulting in increased apoptotic markers (cleaved PARP, caspase-3/7 activation), accumulation of γH_2_AX, and marked suppression of HR (RAD51) and NHEJ (Ku80) proteins [226,227]. Importantly, in melanoma and cisplatin-resistant urothelial carcinoma models, pretreatment with VPA or quisinostat effectively restored sensitivity to DTIC and talazoparib, underscoring the capacity of HDACi to reverse both intrinsic and acquired drug resistance [226,228].

The therapeutic relevance of this combination strategy has been further validated in vivo. In an ex vivo lung metastasis model derived from Ewing sarcoma cells, kt-3283 significantly inhibited tumor cell colonization at concentrations as low as 10 nM [224]. In patient-derived SCLC xenograft models, combined treatment with CUDC-907 and olaparib achieved substantially greater tumor growth inhibition than either monotherapy, accompanied by reduced Ki67 staining, increased cleaved caspase-3, and downregulation of RAD51 and Ku80 within tumor tissues [226]. In melanoma NSG mouse xenografts, VPA in combination with DTIC and talazoparib similarly reduced tumor burden [228]. Notably, certain HDACi, particularly quisinostat, preferentially induced a reversible senescence-like state in normal urothelial epithelial cells rather than overt cytotoxicity, suggesting a potentially favorable therapeutic window for HDACi–PARPi combination regimens [227]. In prostate cancer models, veliparib and vorinostat degrade the UHRF1/BRCA1 complex, reducing DU145 xenograft tumor volume by 65% (*p* < 0.01) [230].

As a single molecular entity, the pharmacokinetic properties of these inhibitors are more manageable, reducing potential interference caused by differences in drug metabolism pathways during combination therapy [222,223]. For instance, vorinostat, through its deacetylation activity, activates EZH2, inhibiting the HR pathway, which sensitizes BRCA wild-type tumors to olaparib [227]. In addition, HDAC inhibitors enhance PARP1’s DNA trapping capability, increasing γH2AX foci by 2.3-fold in TNBC models [228]. Preclinical studies show that the combination of panobinostat with talazoparib significantly reduces cell viability (70% reduction, CI = 0.32), effectively inhibiting the ATM/BRCA1 pathway in pancreatic cancer cell lines [229].

#### 5.1.2. PARPi and Cyclin-Dependent Kinase Inhibitors (CDKi) Combination

Recent studies indicate that combining PARPi with CDKi yields synergistic anti-tumor effects across various cancer models [231,232,233,234]. The core mechanism of this combination lies in the accumulation of SSBs induced by PARP inhibition, which subsequently leads to replication fork collapse and DSB formation [231,235]. Meanwhile, CDKi interfere with the phosphorylation of key proteins involved in HRR (e.g., BRCA1, RAD51), thus amplifying DNA damage accumulation while blocking HRR [235,236]. For example, in BRCA1-mutant, RB-deficient TNBC cells (HCC1937), the combination of abemaciclib and talazoparib significantly reduces cell viability (to 45.9% in the combination group, compared to 70.8% with abemaciclib alone) [232]. Further analysis revealed that this combination induced a significant G0/G1 phase arrest (78.4% vs. 58.3% in the control group) and upregulated apoptotic proteins such as Bax (1.8-fold) and Caspase-3 (5.3-fold), indicating that this combination induces both intrinsic and extrinsic apoptotic pathways to exert its synergistic anti-tumor effect [232].

In a multiple myeloma model, dinaciclib inhibits phosphorylation of the BRCA1 Ser1497 site, blocking RAD51 foci formation and reducing HR repair efficiency by 84% (*p* < 0.0001), leading to synthetic lethality [237]. Mechanistically, CDK inhibitors can interfere with the transcription of RAD51 and its homologous genes (e.g., XRCC3), resulting in decreased RAD51 protein levels and enhancing the cytotoxicity of PARPi like ABT-888, which has shown significant effects in myeloma cells (*p* < 0.01), though no similar effects were observed in normal CD19^+^ B cells [237].

The synergistic effects of combination therapy have been validated in multiple cancer types. In acute myeloid leukemia (AML) cells (MV4-11), a novel dual CDK9/PARPi (compound **34**) exhibits IC_50_ values of 29 nM for CDK9 and 10 nM for PARP1, showing broad anti-proliferative effects in ovarian cancer, TNBC, and bladder cancer cell lines (IC_50_ range: 0.13–2.52 μM) [233]. This mechanism includes dose-dependent reduction in downstream effectors such as p-RNAP II (Ser2) and c-MYC expression, along with enhanced efficacy through G2/M phase arrest and migration inhibition in MDA-MB-231 cells [233]. Notably, in BRCA wild-type but HR-deficient models, CDK4/6 inhibitors, though ineffective alone, can overcome resistance when combined with PARPi. In HCC1937 cells, the combination of abemaciclib and talazoparib increased apoptosis rates from 17.9% (single agent) to 59.2%, and this effect was reproduced in in vivo mouse xenograft models, with delayed tumor growth nearly two-fold (*p* < 0.01) and significantly extended survival (P < 0.004) [232]. This strategy achieves selective tumor cell killing while minimizing effects on normal cells by disrupting multiple DNA damage repair pathways simultaneously [232]. Moreover, dual-target inhibitors (e.g., CDK9/PARPi) optimize pharmacokinetics (e.g., liver microsomal half-life T_1/2_ = 2.44 h) and improve the therapeutic window, presenting potential for clinical translation.

#### 5.1.3. PARPi and Androgen Receptor Signaling Inhibitors (ARSIs) Combination

For patients with newly diagnosed metastatic castration-resistant prostate cancer (mCRPC), the combination of PARPi with ARSIs has emerged as a profound first-line treatment option, irrespective of their HRR gene status. Clinical trials have demonstrated significant benefits in both the overall population and the HRR-mutated subgroup. The combination of PARPi with ARSIs capitalizes on the bidirectional crosstalk between DDR and AR signaling pathways [238,239,240]. Inhibition of AR downregulates the transcription of HRR genes, such as BRCA1/2, RAD51, and PALB2, thus inducing a functional “BRCAness” phenotype that sensitizes tumors to PARPi treatment [240,241]. Conversely, PARP1 serves as a coactivator for AR-mediated transcription, and its inhibition suppresses AR-driven gene expression programs, including those involved in DDR [241,242]. Preclinical studies have demonstrated that AR blockade increases PARP activity, which further enhances the efficacy of PARPi [243]. This synthetic lethality is particularly potent in tumors that exhibit co-loss of RB1 and BRCA2, a genetic alteration found in up to 50% of mCRPC, and is a driving factor of aggressive disease progression [244]. Additionally, the incorporation of PARPi may mitigate resistance mechanisms mediated by AR splice variants (AR-V7) or the loss of RB1, overcoming adaptive resistance to ARSIs (Figure 6) [237,244].

In vitro studies have consistently shown synergistic cytotoxicity when PARPis are combined with ARSIs. Enzalutamide treatment suppresses HRR gene expression and increases γH2AX foci (a marker of DNA DSBs), while olaparib inhibits PARP1-mediated DNA repair, leading to the accumulation of unrepaired DNA damage [245]. Combination treatment with enzalutamide and olaparib results in significantly higher rates of apoptosis (cleaved caspase-3) and clonogenic death compared to monotherapy in AR-positive prostate cancer cell lines (LNCaP, VCaP) [242,246]. In vivo xenograft models confirm these findings, with the enzalutamide plus olaparib combination inducing greater tumor regression and prolonged survival than either agent alone, particularly in models with functional HRR pathways [242]. Notably, a lead-in strategy in which enzalutamide pretreatment precedes olaparib administration maximizes HRR gene suppression and enhances PARPi-induced DNA damage, resulting in superior antitumor efficacy [242].

Phase III clinical trials have validated the efficacy of PARPi-ARSI combinations in mCRPC. The PROpel trial (olaparib + abiraterone) demonstrated a significant improvement in radiographic PFS across all patient cohorts (HR 0.66; 95% CI 0.54–0.81), with the greatest benefit observed in BRCA1/2-mutated patients (HR 0.23; 95% CI 0.12–0.43) [247]. The MAGNITUDE trial (niraparib + abiraterone) confirmed rPFS benefit exclusively in HRR-deficient cohorts (HR 0.76 for HRRm; HR 1.09 for non-HRRm), leading to the early termination of the non-HRRm arm [248]. In contrast, the TALAPRO-2 study (talazoparib + enzalutamide) showed rPFS improvement in both HRR-deficient (HR 0.45) and non-HRR-deficient (HR 0.70) populations, suggesting broader applicability [249]. A pooled analysis of these trials indicates a clear hierarchy of benefit: BRCA1/2 alterations confer the greatest magnitude of benefit (pooled HR 0.32), followed by any HRR mutation (pooled HR 0.55), with the lowest benefit observed in non-HRRm patients (pooled HR 0.74) [245]. Safety profiles reveal increased hematologic toxicity with combination therapy, particularly anemia (grade ≥3 in 46% of talazoparib-treated patients), though dose reductions mitigate discontinuation rates [249].

#### 5.1.4. PARPi and RNF168 or RAD52 Inhibitors Combination

Although BRCA1-deficient cells are prone to loss of HR function, DNA end resection can still occur in these cells, suggesting that BRCA1’s role in HR extends far beyond promoting resection [19]. Recent work confirms that BRCA1 facilitates the assembly of BRCA2-dependent RAD51 nucleoprotein filaments by recruiting the PALB2–BRCA2 complex to ssDNA, a step essential for HR completion [250,251]. In the absence of BRCA1, PALB2 can be recruited to ssDNA via RNF168, explaining how HR reactivation in BRCA1/53BP1 double-deficient cells depends on RNF168-mediated PALB2 recruitment and on enhanced end resection following loss of the 53BP1–RIF1–REV7–shieldin axis (Figure 6) [19,251]. The degree of HRR depends on the BRCA1 mutation type, as hypomorphic alleles retaining PALB2-binding capacity substantially improve RAD51 loading and thereby increase PARPi resistance [252]. Consequently, inhibiting RNF168 to block this BRCA1-independent PALB2/BRCA2 recruitment may resensitize BRCA1-mutant tumors that have acquired resistance through 53BP1 pathway inactivation [43]. Meanwhile, the DNA repair protein RAD52 has attracted growing interest as an alternative HR mediator [253]. While early studies reported minimal effects of RAD52 loss on viability, subsequent work revealed that RAD52 knockout is synthetically lethal with BRCA1/2 deficiency, suggesting RAD52 provides a backup pathway to recruit RAD51 to resected ends [254,255]. RAD52 also participates in repairing ssDNA at stalled replication forks and may promote fork reversal, implying that its synthetic lethality arises from the combined impact of these functions. Recently developed small-molecule RAD52 inhibitors have shown synergistic cytotoxicity with PARPi against BRCA-deficient cells in preclinical models [256].

### 5.2. PARPi and Immunotherapy Combination

The synergistic effects of PARPi and immune checkpoint inhibitors (ICI) largely depend on the innate immune activation triggered by DNA damage. In BRCA1-deficient tumors, PARPi exacerbates genomic instability by inhibiting SSBR, leading to an accumulation of DSBs and activation of the cGAS-STING pathway (Figure 6) [257,258]. This pathway induces the production of type I interferons and the expression of chemokines (e.g., CXCL10, CCL5), which promote dendritic cell maturation and T-cell infiltration (Figure 6) [259,260]. Notably, PARPi treatment also upregulates PD-L1 expression (inactivation of GSK3β) on tumor cells, creating an adaptive immune suppressive microenvironment, thus providing a theoretical basis for combining PD-L1 blockade (Figure 6) [258]. Studies in small-cell lung cancer (SCLC) further revealed that PARPi combined with radiotherapy downregulates the translation repressor EIF4E2, stabilizing CXCL10 mRNA and enhancing its protein expression, thereby amplifying T-cell recruitment effects [260]. The EIF4E2 regulatory mechanism identified in SCLC provides a new perspective: the combination of PARPi and radiotherapy degrades EIF4E2, relieving its binding to the AU-rich element in the 3′ UTR of *CXCL10* mRNA, thereby extending the mRNA half-life 3–4 folds. Ovarian cancer models show that prolonged PARPi treatment activates STAT3 signaling in tumor cells, inducing the polarization of tumor-associated macrophages toward an M2 pro-tumor phenotype, characterized by a significant increase in the CD163^+^/CD206^+^ cell ratio [259]. This polarization suppresses T-cell function through the secretion of IL-6, CCL2, and other factors, thereby reducing PARPi sensitivity [260]. Another recent study demonstrated that T cells from cancer patients accumulate DNA damage during prolonged PARPi exposure, ultimately impairing therapeutic efficacy. To address this limitation, cytosine base editing (CBE3) was applied to introduce precise point mutations (D45N/M43I, S588F, G745K) into critical DNA-binding interfaces of PARP1 in CAR-T cells, encompassing interdomain regions of the ZnF, WGR, and HD modules [65]. These edits markedly reduced PARPi-induced PARP1 trapping and associated genotoxic stress. As a result, the engineered CAR-T cells displayed enhanced persistence, expansion, and cytotoxic activity under combination treatment conditions. Collectively, this strategy effectively circumvented PARPi-associated resistance, leading to robust tumor suppression and significantly extended survival in preclinical models, thereby providing a novel framework for optimizing PARPi-immunotherapy combinations [65].

Immunological reprogramming of TME is central to the synergistic enhancement of combination therapies. The HRD functional phenotype is closely linked to immunogenicity. In isocitrate dehydrogenase (IDH)-mutant gliomas, the planar cell polarity protein Prickle4 has emerged as a key driver of PARPi-induced neovascularization. Targeting Prickle4 restores PARPi sensitivity, underscoring its role in resistance. Transcriptomic analyses indicate that PARPi not only reshapes the vascular niche but also reprograms the immune microenvironment. Treatment is associated with enhanced pro-inflammatory signaling, increased CD4^+^ and CD8^+^ T-cell infiltration, and upregulation of PD-L1 expression. These changes suggest a shift from an immunologically “cold” phenotype toward a more inflamed tumor state. Accordingly, inhibition of the Prickle4 pathway, combined with anti-angiogenic agents or immunotherapy, may represent a rational strategy to overcome PARPi resistance in IDH-mutant glioma [261]. Combination therapies reverse PARPi resistance and reprogram the immune microenvironment to achieve synergistic antitumor effects [262,263]. The HRD-EXCUTE phenotype upregulates immune chemokines such as CXCL10/11, CCL2/8, and IFNβ1, significantly increasing tumor mutational burden (TMB) and neoantigen load, thus promoting CD8^+^ T-cell, M1 macrophage, and activated dendritic cell infiltration. Patients with this phenotype are more likely to respond to immune checkpoint blockade (ICB) [263]. In contrast, HRD tumors lacking the HRD-EXCUTE phenotype display an immunosuppressive microenvironment, with T-cell exhaustion and M2 macrophage enrichment, leading to resistance to ICB [263]. PARPi combined with HDACi can activate the cGAS-STING pathway and increase H3K27ac enrichment at HRD-EXCUTE loci, further enhancing tumor immunogenicity [263]. Single-cell analysis indicates that LP subtype breast cancer patients, despite genomic instability, exhibit high expression of immune checkpoint molecules (e.g., PD-L1) and show sensitivity to PARPi and ICB combination therapy [264]. These findings emphasize that combination therapy reshapes the immune microenvironment, converting “cold” tumors into “hot” tumors, a critical mechanism for synergistic enhancement.

In advanced endometrial cancer, MMRp patients receiving first-line ICI monotherapy have shown survival benefits, while the subgroup of MMRp patients with p53 mutations (*n* = 590) treated with PARPi + ICI combination therapy displayed a significant reduction in PFS risk (HR = 0.42), outperforming ICI monotherapy (HR = 0.47) or PARPi monotherapy (HR = 0.63) [265]. PD-L1-positive tumor patients (*n* = 1121) derived even greater benefit from combination therapy, with a 13.9% increase in 12-month PFS rate compared to ICI monotherapy (57.6% vs. 43.7%) [265]. In PARPi-resistant BRCA1-mutated breast cancer, high p-Y158 PARP1 levels (IHC H-score > median value) correlate with poor prognosis and can serve as a predictive biomarker for FGFRi combination therapy [266]. The HRD-EXCUTE score in ovarian cancer, as a functional phenotype biomarker, can precisely identify patients sensitive to PARPi + HDACi combination therapy, with a significant increase in the proportion of IFNγ+ CD8^+^ T-cells and M1 macrophages in their tumors [263]. In SLFN11-positive extensive-stage small cell lung cancer (ES-SCLC) patients, a phase II randomized study (S1929) showed that maintenance therapy with talazoparib and atezolizumab significantly improved PFS compared to atezolizumab monotherapy (median PFS: 2.9 months vs. 2.4 months; HR = 0.66). Clinical preclinical studies further revealed that the combined strategy primarily overcomes PARPi resistance through synergistic activation of the cGAS-STING immune pathway, induction of immunogenic cell death, and enhanced infiltration of CD8^+^ T cells into TME, providing a critical mechanistic foundation for overcoming PARPi resistance [267].

### 5.3. PARPi and Ionizing Radiation (IR) Combination

Combination strategies that concurrently suppress DNA repair pathways and reprogram the tumor immune microenvironment markedly potentiate the radiosensitizing effects of PARPi [268,269,270,271]. In the neoadjuvant treatment of locally advanced rectal cancer, a phase Ib clinical trial (NCT01589419) evaluated the efficacy of veliparib combined with capecitabine and radiotherapy (RT) (50.4 Gy) [272]. Among 31 evaluable patients, the regimen demonstrated favorable tolerability and encouraging antitumor activity: 71% (22/31) of patients achieved tumor downstaging, with 29% (9/31) attaining a pathological complete response (pCR). These findings suggest that the combination of a PARPi with chemoradiotherapy may enhance the pCR rate in the preoperative neoadjuvant setting [272]. In patients with solid tumors and brain metastases, a phase I trial (NCT00649207) explored the combination of veliparib with whole-brain radiotherapy (WBRT). The median overall survival in non-small cell lung cancer and breast cancer subgroups reached 10.0 months and 7.7 months, respectively, surpassing the predicted values from historical nomograms (3.5 months and 4.9 months, respectively), indicating that this combined strategy may improve outcomes in this patient population [273].

Preclinical studies further elucidate the cellular and molecular mechanisms underlying this synergy. In inflammatory breast cancer cell models (e.g., SUM-190, MDA-IBC-3), PARPi as monotherapy show limited efficacy, but when combined with RT, even at sub-micromolar concentrations, they produce significant radiosensitization, with radiation enhancement ratios (rER) ranging from 1.12 to 1.76. In the SUM-190 xenograft model, the combination therapy significantly delayed tumor doubling time with manageable toxicity [274]. In TNBC models, the combination of talazoparib with RT can induce therapy-induced senescence. Subsequent administration of a senolytic agent (e.g., Navitoclax) selectively eliminates these senescent cells, thereby significantly promoting apoptosis in both in vitro and in vivo models and overcoming tumor recurrence and resistance mediated by senescence [275].

Mechanistically, the combination therapy enhances efficacy and reverses resistance through multiple pathways. Under oxidative stress conditions, nitric oxide donors can downregulate BRCA1 expression, inhibit high-fidelity HRR, and erroneously promote error-prone NHEJ. This leads to the accumulation of DNA damage and genomic instability, thereby achieving radiosensitization with PARPi even in tumors with functional HRR [268]. In muscle-invasive bladder cancer cells, particularly those with TP53 mutations, the combination of olaparib and RT exhibits significant synergistic cytotoxicity, with dose enhancement factors reaching 1.4 to 2.3-fold. The mechanism involves PARP inhibition increasing mitochondrial reactive oxygen species production, which induces DSBs. Cells with p53/ATM functional loss are more sensitive to such damage, thereby overcoming potential resistance to PARPi [269].

### 5.4. PARPi and Other Combination Therapies

The combination of PARPi with oncolytic herpes simplex viruses (oHSVs) has demonstrated pronounced synergistic antitumor efficacy, particularly in glioblastoma multiforme (GBM). Ning et al. reported that, despite substantial heterogeneity in the intrinsic sensitivity of glioblastoma stem cells (GSCs) to PARPi, oHSV treatment uniformly sensitized both PARPi-responsive and -resistant GSC populations through the selective degradation of key response DDR proteins, most notably RAD51 and CHK1, in a viral replication-dependent manner [276]. Consistent with this observation, in vitro analyses revealed that the combinatorial regimen significantly increased DSBs accumulation, apoptotic signaling, and tumor cell death. Importantly, in orthotopic xenograft models established from patient-derived GSCs, combination therapy resulted in a marked extension of median survival compared with monotherapy. In PARPi-sensitive tumors, median survival reached 131 days with the combination, compared with 83 days following olaparib treatment alone [276]. Similarly, in PARPi-resistant tumors, median survival was prolonged to 75 days, compared with 54 days in mice treated with either olaparib or MG18L monotherapy, without additional toxicity to normal glial cells [276]. Mechanistic investigations indicated that this synergistic effect was not mediated by alterations in viral replication kinetics but rather by disruption of tumor-intrinsic DDR pathways, particularly RAD51-dependent HRR.

At the molecular level, this cooperative interaction arises from the capacity of oHSVs to exert multifaceted interference with DDR signaling networks. Although GSCs exhibit heterogeneous baseline responses to PARP inhibition, oHSV infection consistently induces selective degradation of critical DDR components, including RAD51 and CHK1, in a manner dependent on viral DNA replication. Notably, a genome-wide CRISPR screening approach identified PARP1 as a host restriction factor limiting HSV-1 replication, providing a mechanistic basis for the enhanced oncolytic activity observed upon PARP inhibition [277]. Across both GBM and TNBC models, the PARPi–oHSV combination consistently outperformed either agent alone and was accompanied by increased expression of immune checkpoint molecules on CD4^+^ T cells, suggesting concomitant activation of antitumor immune responses [277]. Collectively, these findings indicate that PARPi–oHSV co-treatment exploits DDR vulnerabilities to overcome PARPi resistance. Importantly, the marked survival benefit observed in tumors derived from both PARPi-sensitive and -resistant GSCs underscores the potential clinical applicability of this strategy across distinct molecular subtypes of GBM.

An emerging therapeutic paradigm combines PARPi with PROTACs to overcome both intrinsic and acquired resistance, supported by extensive in vitro and preclinical evidence. Resistance to PARPi commonly arises from multiple mechanisms, including replication fork stabilization mediated by factors such as NSD3S, as well as point mutations in PARP1 that diminish inhibitor binding. PROTAC-based strategies address these limitations by inducing selective and sustained degradation of resistance-associated proteins, thereby bypassing the constraints of conventional enzymatic inhibition (Table 2) [278,279,280]. Mechanistically, NSD3S has been shown to accumulate at stalled replication forks, where it preserves nascent DNA integrity by antagonizing PTIP-dependent recruitment of the MRE11 nuclease, thus promoting fork stability. This process is driven by ATR-dependent signaling, whereas NSD3S turnover is regulated by the CUL3–ZBTB2 E3 ubiquitin ligase complex. Targeted degradation of NSD3S using PROTACs effectively destabilizes replication forks and restores PARPi sensitivity in resistant tumor models [278].

In TNBC, point mutations in PARP1 frequently undermine the efficacy of classical PARP inhibition. To address this challenge, PROTAC molecules such as **NN3** have been developed to directly eliminate mutant PARP1, thereby triggering ferroptosis and robust tumor cell death, particularly in p53-proficient breast cancer cells. This effect is characterized by suppression of the SLC7A11 antioxidant pathway and accumulation of lipid peroxides, underscoring a distinct, non-apoptotic vulnerability exploitable by PROTAC-based approaches [279]. From a medicinal chemistry perspective, conventional PARPi often exert strong PARP-trapping activity, which can translate into dose-limiting toxicity. In contrast, newly developed benzimidazole-based PARP1 ligands (e.g., 19A10) retain high target affinity (IC_50_ = 4.62 nM) while exhibiting reduced PARP-trapping capacity, thereby providing an improved scaffold for the design of safer and more effective PARP1-directed PROTACs [280].

Consistent with these mechanistic insights, both in vitro and in vivo studies demonstrate that NSD3-directed PROTACs markedly enhance PARPi responsiveness in cell line-derived xenografts and PDX models. Similarly, PARP1-targeting PROTACs display potent antitumor activity with favorable toxicity profiles in PARPi-resistant mouse models [278,279]. Collectively, these findings establish an innovative “inhibition–degradation” combinatorial strategy, in which PROTAC-mediated protein clearance complements pharmacological PARP inhibition to overcome resistance. This approach holds promise not only for HRD tumors but also for HR-proficient malignancies that acquire PARPi resistance through noncanonical mechanisms [278,279,280]. Moreover, emerging evidence suggests that activation of the MMEJ pathway, driven by POLQ, may contribute to BRCA2 or PALB2 reversion mutations in PARPi-resistant tumors. Accordingly, co-targeting PARP and POLQ represents a rational strategy to suppress this compensatory repair pathway, although its clinical efficacy remains to be established (Figure 6) [281,282].

Another combination strategy recently investigated is PARPi with NAT10 inhibitors. The N4-acetylcytidin (ac4C) modification catalyzed by NAT10 on DNA:RNA hybrids reinforces their structural integrity, which in turn augments HRR efficiency [283]. This NAT10-dependent pathway constitutes a significant mechanism contributing to resistance against PARPi. Preclinical studies in hepatocellular carcinoma, ovarian cancer, and breast cancer models, both in vitro and in vivo, have demonstrated that co-treatment with the NAT10 inhibitor remodelin and olaparib produces synergistic antitumor effects, effectively overcoming PARPi resistance [284]. Mechanistically, remodelin functions by suppressing the acetyltransferase activity of NAT10. This inhibition leads to the destabilization of DNA:RNA hybrids, consequent impairment of HR repair, and ultimately establishes a synthetic lethal relationship with the DNA damage repair blockade induced by PARPi [284].

**Table 2:** Combination therapies strategies for overcoming PARPi resistance.

Combination Therapy	Treatment	Tumor Type	Overcoming PARPi Resistance Mechanisms ^1^	Safety ^2^	Ref
PARPi-HDACi Combination	Olaparib + Vorinostat	PC	a	DU145 tumor xenograft mice: (-)	[223]
PARPi-HDACi Combination	bifunctional PARPi (**kt-3283**)	ES	a	Animal studies: ND	[224]
PARPi-HDACi Combination	Olaparib + **CUDC-907**	SCLC	a	PDX models of SCLC: (-)	[226]
PARPi-HDACi Combination	Talazoparib + Quisinostat	UC	a	Animal studies: ND	[227]
PARPi-HDACi Combination	Talazoparib + Valproic acid	TNBC	a	Animal studies: ND	[228]
PARPi-HDACi Combination	Talazoparib + Panobinostat + Decitabine	BC, OV	a	Animal studies: ND	[229]
PARPi-HDACi Combination	Veliparib + Vorinostat	PC	a	DU145 tumor xenograft mice: (-)	[230]
PARPi-CDKi Combination	Rucaparib + CRISPR/Cas9-mediated inactivation of CDK12	PC	b	*CDK12* KO tumor xenografts: (-)	[231]
PARPi-CDKi Combination	Talazoparib + Abemaciclib	TNBC	b	Animal studies: ND	[232]
PARPi-CDKi Combination	Dual CDK9/PARPi **36**	TNBC	b	Animal studies: ND	[233]
PARPi-CDKi Combination	Olaparib + Fadraciclib	BLBC	b	BRCA1 mutant BLBC patient derived xenograft models: (-)	[235]
PARPi-CDKi Combination	Olaparib + Dinaciclib	TNBC	b	PDX 127 xenograft model: (-)	[236]
PARPi-ARSIs Combination	Olaparib + Enzalutamide	mCRPC	c	MDA 133-4 PDX model: (-)	[242]
PARPi-ARSIs Combination	PROTAC (**BSJ-5-63**) + Olaparib	CPRC	c	AR- positive 22Rv1 xenograft model: (-)	[72,244]
PARPi-ARSIs Combination	Olaparib + Abiraterone	CRPC	c	PROfound (phase III). The incidence of anemia in the combination therapy group was 50%, with grade ≥3 anemia accounting for 16%; in the control group (vehicle + abiraterone), the corresponding rates were 18% and 3%, respectively. In the combination group, 18% of patients required at least one blood transfusion.	[245]
PARPi-ARSIs Combination	Niraparib + AAP (Abiraterone Acetate + Prednisone)	mCRPC	c	MAGNITUDE (NCT03748641, phase III). This combination increased the incidence of hematological toxicities, particularly anemia. However, with proactive monitoring and management (e.g., dose adjustments), its safety profile was manageable, no new safety signals emerged, and the benefit-risk ratio remained favorable.	[248]
PARPi-ARSIs Combination	Talazoparib + Enzalutamide	mCRPC	c	TALAPRO-2 (NCT03395197, phase III). Hematologic events—primarily anemia—being the most common adverse effects. With careful monitoring, especially during the first 3–4 months, and timely dose adjustments with supportive care, most patients were able to continue treatment.	[249]
PARPi-Immunotherapy Combination	Olaparib + CAR T therapies	EOC	d	EOC PDX models: (-)	[65]
PARPi-Immunotherapy Combination	Olaparib + STING agonist	OV	d	Animal studies: ND	[260]
PARPi-HDACi-Immunotherapy Combination	Niraparib/Olaparib + Entinostat + α-PD-1	OV	d	Animal studies: ND	[263]
PARPi-Immunotherapy Combination	Atezolizumab + Talazoparib	Schlafen 11 SLFN11-ES-SCLC	d	S1929 (phase II). It was found that hematological toxicity was significantly increased in this combination group, whereas non-hematological toxicity was similar. The rate of treatment discontinuation due to toxicity was low.	[267]
PARPi + IR Combination	Veliparib + DETA	NSCLC	e	Animal studies: ND	[268]
PARPi + IR Combination	Veliparib + IR	LARC	e	NCT01589419, phase Ib. Nausea, diarrhea, and fatigue were the most frequent adverse events, with limited grade 3–4 toxicity. Pharmacokinetic analysis showed dose-proportional veliparib exposure and no significant interaction with concomitant chemotherapy.	[272]
PARPi + IR Combination	Veliparib + whole brain radiation therapy (WBRT)	NSCLC, BC	e	NCT00649207, Phase I. The most common toxicities were fatigue, nausea, and decreased appetite, while severe treatment-related adverse events were rare.	[273]
PARPi + IR Combination	Olaparib + IR	IBC	e	SUM-190 IBC mouse xenograft model: (-)	[274]
PARPi + IR Combination	Talazoparib + IR	TNBC	e	Animal studies: ND	[275]
PARPi + oHSVs Combination	Olaparib + MG18L or G47D	GBM	f	Mice bearing PARPi-sensitive and-resistant GSC-derived intracerebral tumors: (-)	[276]
PARPi + oHSVs Combination	Olaparib + Oncolytic viruses (SH100)	GBM, TNBC	f	BALB/c mice bearing 4T1: (-)	[277]
PARPi + PROTAC Combination	Olaparib + NSD3 PROTAC	mCRPC	g	PR10-derived mCRPC PDX models: (-)	[278]
PARPi + PROTAC Combination	**NN3** PROTAC	TNBC	g	postsurgical BALB/c nude mouse model bearing MDA-MB-231 xenografts: (-)	[279]
PARPi + PROTAC Combination	**19A10** PROTAC	TNBC	g	Animal studies: ND	[280]
PARPi + ATRi Combination	Talazoparib or olaparib + **VE-821**	SCLC	h	Animal studies: ND	[45]
PARPi + POLQi Combination	Olaparib + **NCT-505**	OV	i	Animal studies: ND	[281]
PARPi + POLQi Combination	Olaparib + **ART558**	BC	i	Animal studies: ND	[282]
PARPi + NAT10i Combination	Olaparib + Remodelin	HCC	j	NAT10-knockout mouse model: (-)	[284]

^1^ The alphabet (a–j) of overcoming PARPi resistance mechanisms section represents the classification of combination therapy mechanisms in Figure 5. ^2^ (-), No significant toxicity was observed in animal studies; ND, animal testing not detected. Bold text represents novel compounds reported in the references.

## 6. Conclusions and Future Perspectives

PARPi have transformed the therapeutic landscape of HRD malignancies by exploiting synthetic lethality within the DDR network. The catalytic inhibition of PARP1 and stabilization of PARP–DNA complexes convert unrepaired single-strand breaks into cytotoxic double-strand breaks, thereby selectively eliminating tumors harboring BRCA1/2 alterations. Beyond direct DNA repair blockade, PARPis also exert immunomodulatory effects through activation of the cGAS–STING pathway, induction of PD-L1 expression, and remodeling of the tumor immune microenvironment, providing a mechanistic basis for rational combination strategies.

Despite these advances, acquired resistance remains the principal obstacle to durable clinical benefit. Resistance arises through coordinated adaptive mechanisms, including restoration of HRR via BRCA reversion mutations or disruption of the 53BP1–Shieldin axis, re-establishment of replication fork protection, metabolic rewiring that sustains DNA repair capacity, and pharmacodynamic alterations such as enhanced drug efflux or reduced PARP1 trapping. These interconnected processes indicate that PARPi resistance reflects dynamic systems-level reprogramming rather than isolated molecular events.

A primary approach builds upon synthetic lethality by co-targeting complementary DNA damage repair pathways or reinforcing the “BRCAness” phenotype. Inhibitors of ATR, CDK12, WEE1, and CDK4/6 have shown the capacity to augment PARPi-induced cytotoxicity and exhibit activity in BRCAness-like tumors or PARPi-resistant models. Dual inhibition of PARP and HDAC further represents a mechanistically compelling strategy. Beyond modulating chromatin accessibility and DDR gene expression, HDAC inhibition may activate the cGAS–STING pathway, reverse immunosuppressive signaling, and promote synergy between targeted therapy and immune activation.

Metabolic co-targeting provides an additional avenue to intensify synthetic lethality. Agents such as NAMPT inhibitors or PARG inhibitors can exacerbate NAD^+^ depletion or prolong PARP trapping, thereby amplifying metabolic stress and overcoming resistance driven by compensatory pathway activation or metabolic adaptation. In the context of acquired resistance, inhibition of alternative DNA repair mediators—including POLQ or RAD52—has emerged as a promising strategy to target backup repair pathways exploited by BRCA-deficient tumors. The integration of PARPi with immune checkpoint inhibitors is particularly attractive. PARPi-induced DNA damage promotes cytosolic DNA accumulation and STING activation, enhancing tumor antigenicity and antitumor immune responses. Early-phase clinical studies in gynecologic malignancies and SCLC suggest encouraging activity of PARPi–immunotherapy combinations.

Nevertheless, combination regimens introduce additional safety considerations. Kinase inhibitors such as ATR or CDK12 inhibitors may exacerbate myelosuppression and gastrointestinal toxicity. PARPi–HDACi combinations are frequently associated with hematologic adverse events and fatigue, whereas immune combinations increase the risk of immune-related toxicities, including pneumonitis, colitis, and hepatitis. Moreover, adaptive upregulation of drug transporters such as ABCB1 may reduce intratumoral drug accumulation and contribute to multidrug resistance.

Therapeutic efficacy is additionally constrained by limited drug accessibility and efflux-mediated resistance, underscoring the importance of advanced delivery systems. Nanoparticles, liposomes, and engineered extracellular vesicles enable tumor-selective accumulation and co-delivery of sensitizing agents, potentially improving intratumoral penetration while minimizing systemic toxicity. Such platforms may be particularly relevant in central nervous system malignancies, where blood–brain barrier permeability restricts effective drug exposure. The integration of artificial intelligence and large-scale multi-omics analysis provides a powerful framework to decipher resistance heterogeneity, identify non-canonical regulatory nodes, and optimize rational combination design. Computational modeling may accelerate the discovery of novel synthetic lethal interactions and facilitate the development of next-generation PARPi with improved pharmacologic properties and reduced susceptibility to efflux transporters.

In summary, the clinical application of PARPi has evolved from single-pathway inhibition toward an integrated paradigm that combines DDR targeting, immune modulation, metabolic intervention, and emerging technologies. Future progress will depend on systems-oriented strategies that integrate mechanistic biology, innovative delivery platforms, and data-driven precision oncology. Such an approach holds promise for extending response durability and broadening the clinical impact of PARPi across diverse tumor contexts.

## Figures and Tables

**Table 1:** The development status of current and next-generation PARPi.

Drugs	Chemical Structures	Target	Cancer Type	IC_50_ (nM)	T_1/2_ (h)	Clinical Stage	Side Effects (abcdef)	Overall Efficacy	Ref
Olaparib	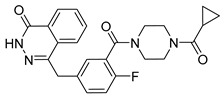	PARP1/2	BC, OC, PC	5.0, 1.0	11–14	III/IV	a, b	OC: Median PFS: 56 mo (olaparib) vs. 13.8 mo (placebo)	[57,58]
Niraparib	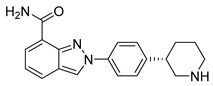	PARP1/2	MPC, OC, PC	3.8, 2.1	36	III/IV	c, d	OC: Median PFS: 18.3 mo (niraparib) vs. 5.4 mo (placebo)	[75,76]
Senaparib	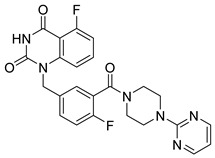	PARP1/2	BC, OC, PC	0.48, 1.6	9	III/IV	a, b, e	OC: Median PFS: >40 mo (senaparib) vs. 13.6 mo (placebo)	[77]
Talazoparib	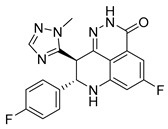	PARP1/2	Advanced BC, SCLC, PC	1.2, 0.87	90	III/IV	a, e, f	Advanced BC: Median PFS: >40 mo (talazoparib) vs. 13.6 mo (chemo); ORR: 62.6% (talazoparib) vs. 27% (chemo)	[78,79]
Fluzoparib	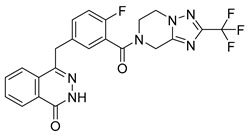	PARP1	OC, Her-2 NBC, BC, RCC	1.46	9	III/IV	a, b, e.	OC: Median PFS: 12.9 mo (fluzoparib) vs. 5.4 mo (placebo)	[80]
Simmiparib	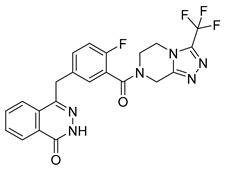	PARP1/2	OC	1.75, 0.22	NA	I	NA	NA	[82]
Rucaparib	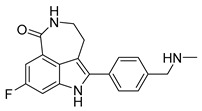	PARP1	mEC, EOC, PC, BC, SCLC	1.0	26	II/III	a, b	EOC: Median PFS: 20.3 mo (rucaparib) vs. 9.1 mo (placebo)	[83,84]
Pamiparib	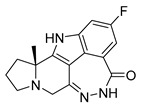	PARP1/2	GBM, OC, CCPRN	0.9, 0.5	13	III/IV	a, e, f	OC: Median PFS: 15.2 mo (pamiparib); ORR: 64.6%	[85]
Stenoparib	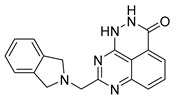	PARP1/2	MBC, Advanced OC	2.0, 1.0	NA	II	NA	OC: Median PFS: >25 mo (stenoparib) vs; 11.5–13 mo (chemo)	[86]
Veliparib	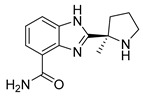	PARP1/2	OC, Recurrent SCLC, mTNBC, BC, PCa	5.2, 2.9	5.2	III	a, b	BC: Median PFS: 25.7 mo (veliparib) vs; 14.6 mo (placebo)	[87,88]
Nesuparib	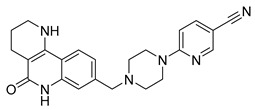	PARP1/2	PDAC, OC	2.0, 2.0	NA	II	NA	NA	[89]
Palacaparib	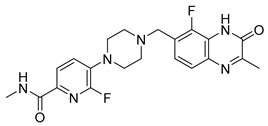	PARP1	BC, advanced malignancies and solid tumor	0.3–2	NA	I/II	a, b, e	NA	[69,90,91]
Mefuparib	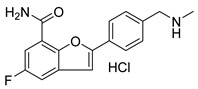	PARP1/2	mCRPC, CCA, BC	1.1, 0.9	NA	II	a	NA	[93]
Venadaparib	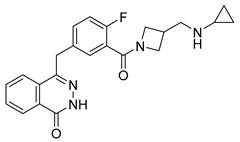	PARP1/2	OV	1.4, 1.0	7–13	I/II	a, b, e	NA	[94,95]
AZD2461	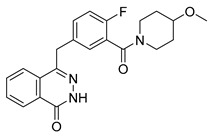	PARP1/2/3	BC, PC	5.0, 2.0, 200	NA	I	NA	NA	[96]
Saruparib	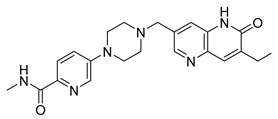	PARP1	BC, OC, PC, CA	3	NA	III	b	BC: Median PFS: 9.1 mo (saruparib); ORR: 48.4%	[92,98]
Compound **33**	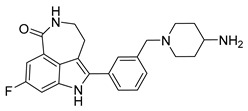	PARP1/2	BC	0.96, 61.9	NA	NA	NA	NA	[97]
Compound **34**	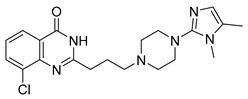	PARP1	BC	9	NA	NA	NA	NA	[98]

IC_50_, The half-maximal inhibitory concentration (nM) of PARPi against their corresponding targets; T_1/2_, half-life (hours); Side effects (abcdef), (a, Gastrointestinal reactions (diarrhea, nausea, vomiting); b, Cytopenias (leukopenia, anemia); c, Bone marrow suppression; d. Allergic reactions (rash, difficulty breathing); e, Fatigue; f, Headache); mo, month; median PFS, median progression-free survival; ORR, objective response rate.

## Data Availability

No new data were created or analyzed in this study.

## Abbreviations

Androgen receptor signaling inhibitors, ARSIs; Breast cancer, BC; Cervical cancer, CA; Cholangiocarcinoma, CCA; Clear cell papillary renal neoplasm, CCPRN; Cyclin-dependent kinase inhibitors, CDKis; DNA damage response, DDR; Double-strand breaks, DSBs; Epithelial ovarian cancer, EOC; Ewing sarcoma, ES; Glioblastoma, GBM; Histone deacetylase inhibitors, HDACIs; Homologous recombination, HR; Homologous recombination deficiency, HRD; Immune checkpoint inhibitors, ICIs; Ionizing Radiation, IR; Locally advanced rectal cancer, LARC; Metastatic breast cancer, MBC; Non-homologous end joining, NHEJ; Metastatic castration-resistant prostate cancer, mCRPC; Metastatic triple-negative breast cancer, mTNBC; Metastatic endometrial cancer, mEC; Metastatic pancreatic cancer, MPC; Ovarian cancer, OC; Oncolytic herpes simplex viruses, oHSVs; Objective response rate, ORR; Overall survival, OS; PARP inhibitors, PARPis; Pancreatic Cancer, PC; Prostate cancer, PCa; Pancreatic ductal adenocarcinoma, PDAC; Progression-free survival, PFS; Renal cell carcinoma, RCC; Recurrent lung small cell carcinoma, Recurrent SCLC; Single-strand breaks, SSBs; Single-strand break repair, SSBR; Tumor microenvironment, TME; Urothelial carcinoma, UC.
